# A scalable outpatient framework for BI-RADS 2–4B breast nodule management: an iodine–DMSO transdermal protocol

**DOI:** 10.3389/frhs.2026.1883180

**Published:** 2026-09-11

**Authors:** João Francisco Pollo Gaspary, Luis Felipe Dias Lopes, Fernanda Peron Gaspary, Carmen Brum Rosa, Antonio Geraldo Camara

**Affiliations:** 1 Institute AuBento—Clinical Practice, Education and Research, Santa Maria, Brazil; 2 Center for Social and Human Sciences, Postgraduate Program in Administration and Accounting, Federal University of Santa Maria, Santa Maria, Brazil; 3 Area of Technological Sciences, Franciscan University, Santa Maria, Brazil; 4 Production Engineering Department, Federal University of Santa Maria, Santa Maria, Brazil; 5 Institute Camara - Center for Clinical and Orthomolecular Practice, Ribeirão Preto, Brazil

**Keywords:** BI-RADS 2–4B, DMSO, functional responsiveness, iodine, outpatient implementation, physiology-guided modulation, transdermal delivery, translational medicine

## Abstract

**Background:**

Low- to intermediate-suspicion breast nodules (BI-RADS 2–4B) generate diagnostic uncertainty, prolonged surveillance, and emotional and operational burden, often without established low-risk interventions designed to support short-interval physiological modulation. Given the redox, endocrine, vascular, and microenvironmental dependencies of benign breast lesions, physiology-guided outpatient modulation may contribute to response-oriented decision-making before conventional oncologic escalation.

**Methods:**

A structured translational outpatient protocol was developed through mechanistic evidence synthesis, multicriteria translational prioritization, protocol structuring, and exploratory clinical implementation. The workflow integrated Evidence-Informed Design, Work Breakdown Structure, multicriteria decision analysis (MCDA), and DesignThinking/Open Innovation. Systematic reviews of molecular iodine and dimethyl sulfoxide (DMSO) identified translational response domains and mechanistic convergence pathways associated with tissue accessibility, redox modulation, and outpatient feasibility.

**Results:**

The translational synthesis identified redox modulation, vascular responsiveness, microenvironmental stabilization, tissue accessibility, and immune-related physiological modulation as principal mechanistic domains associated with iodine-responsive breast-tissue modulation. Comparative prioritization identified the transdermal route as the most operationally feasible outpatient configuration. The finalized protocol—2.5 mL of Lugol's solution (5% iodine) combined with 2.5 mL of pharmaceutical-grade DMSO (99.9%), applied transdermally twice weekly for five weeks—was implemented in three exploratory BI-RADS 4B cases using short-interval MRI and ultrasound reassessment. Two cases demonstrated complete radiologic resolution accompanied by vascular-signal normalization and stromal remodeling, including simultaneous remodeling of contralateral BI-RADS 2 nodules; one remained lesion-free during 12-month follow-up. A third case demonstrated a partial imaging-response pattern and was referred for fine-needle aspiration according to predefined reassessment criteria. No major adverse effects or tolerability-related discontinuations occurred; the intervention was discontinued after six sessions in the third case according to the predefined imaging-based stopping rule.

**Discussion:**

The framework integrates physiological modulation with response-oriented imaging reassessment while preserving compatibility with conventional oncologic workflows. Its low-complexity and reproducible outpatient architecture may possess translational relevance within resource-sensitive and Value-Based Healthcare-oriented breast-care settings.

**Conclusions:**

The exploratory implementation of the iodine–DMSO transdermal protocol yielded biologically coherent, imaging-detectable, and operationally reproducible response patterns compatible with short-interval physiological modulation. These findings support controlled outpatient and implementation-oriented investigations evaluating reproducibility, scalability, cost impact, and broader translational applicability.

**Systematic Review Registration:**

https://www.crd.york.ac.uk/PROSPERO/view/CRD420251122511, PROSPERO CRD420251122511; https://www.crd.york.ac.uk/PROSPERO/view/CRD420251123805, PROSPERO CRD420251123805.

## 1 Introduction

Breast nodules classified within Breast Imaging Reporting and Data System (BI-RADS) categories 2–4B represent a heterogeneous spectrum of lesions that are predominantly benign yet clinically disruptive, capable of triggering diagnostic cascades, prolonged surveillance, and considerable patient anxiety (1, 2). BI-RADS was first introduced to standardize mammographic reporting, terminology, and management recommendations (3) and was later expanded to ultrasonography and MRI in subsequent editions (4). While the BI-RADS system standardized descriptive radiology and improved diagnostic communication, it left an operational and clinical management gap in the intermediate categories, where morphological data alone fail to predict biological behavior (5, 6).

The biological behavior of breast nodules reflects a complex interplay among hormonal signaling, redox balance, and the structural dynamics of the mammary epithelium, in which subtle dysregulations can influence extracellular matrix remodeling, local inflammation, and epithelial proliferation (7–10). Even in the absence of overt malignant imaging features, persistent microenvironmental dysregulation—including stromal activation, extracellular-matrix remodeling, and immune-trajectory divergence—has been documented in benign breast lesions, suggesting that early physiological modulation may represent a clinically actionable strategy before conventional oncologic thresholds are reached (10–12).

Conventional management prioritizes periodic imaging follow-up—typically every six to twelve months—in the absence of rapid growth or suspicious features (D'Orsi et al., 2013; Berg et al., 2012) (4, 13). Although conservative and safe, this passive strategy extends the period in which the nodular microenvironment remains physiologically active yet untreated, forfeiting the opportunity for early modulation that could either promote resolution or reveal non-responsiveness, thereby refining decision-making before oncological thresholds are reached. From a public-health standpoint, this prolonged “watchful waiting” paradigm perpetuates patient anxiety, increases imaging demand, and adds to the cumulative economic burden of benign-disease surveillance in women's healthcare systems worldwide (14, 15).

Diagnostic evaluation traditionally relies on a structured combination of patient history, targeted physical examination, and age-appropriate imaging, often complemented by fine-needle aspiration (FNA) when indicated (16–18). The so-called diagnostic triad—clinical breast examination, imaging, and cytology—yields high diagnostic accuracy when results are concordant. Yet, in cases of discordance or persistent uncertainty from either physician or patient, open surgical biopsy remains the definitive approach (19). This process, although necessary for oncologic safety, creates a persistent operational tension between timely escalation and the avoidance of unnecessary invasive procedures, repeated imaging, and prolonged diagnostic uncertainty. Recent analyses of ultrasound-based BI-RADS subcategories reinforce this diagnostic uncertainty. In a large cross-sectional study of 975 examinations, Spinelli Varella et al. (2018) (5) demonstrated that while overall BI-RADS ultrasonography achieved a discriminating accuracy of 91%, the positive predictive value for malignancy in subcategories 4A and 4B remained low—6% and 25%, respectively—indicating that these subcategories are clinically unfit for screening and do not reliably guide the decision to biopsy. Such findings underscore the persistent gray zone within BI-RADS 2–4B management, where the investigation of structured physiological interventions incorporating short-interval response assessment may provide complementary information for outpatient decision-making, while preserving established biopsy-based diagnostic pathways and oncologic vigilance. Such findings underscore the persistent gray zone within BI-RADS 2–4B management, where a structured physiological intervention capable of differentiating responsive from non-responsive lesions could meaningfully reduce unnecessary invasive procedures and resource use. In tropical or resource-limited settings, where delayed presentation is frequent, the differential diagnosis may also include benign inflammatory lesions or infectious processes—such as abscesses associated with HIV infection (20)—further compounding diagnostic ambiguity and resource strain. This global variability reveals a shared public-health challenge: enabling timely detection of malignancy while simultaneously preventing overtreatment in benign conditions (21).

Beyond clinical uncertainty, the psychological impact of indeterminate lesions is substantial. Patients with benign breast disease (BBD) frequently present higher levels of anxiety and depression than healthy controls (22), as measured by validated instruments such as the Hospital Anxiety and Depression Scale (HADS) and the Brief Patient Health Questionnaire (BPHQ) (23). This emotional burden peaks between initial detection and diagnostic clarification, improving only after confirmation or resolution. Persistent diagnostic ambiguity—particularly in low-suspicion nodules—can erode quality of life, amplify perceived health risks, and impair adherence to medical recommendations, underscoring the relevance of modern multidisciplinary strategies aimed at balancing clinical safety with patient well-being (24).

From a value-based-care perspective, such psychosocial costs translate into measurable reductions in perceived health value and increased utilization of diagnostic resources (25). Therefore, there is a need to investigate safe, low-cost, physiology-guided outpatient strategies capable of addressing local pathophysiology while providing earlier and more tangible clinical feedback. Recent interventional approaches underscore this translational shift, as minimally invasive techniques such as ultrasound-guided high-intensity focused ultrasound (US-HIFU) have demonstrated sustained volumetric, pain, and palpation reduction in fibroadenoma patients over a five-year follow-up (26). Such findings reinforce the notion that benign nodules are not merely structural anomalies but dynamic bioactive systems amenable to physiological modulation.

Within this context, iodine-based physiological modulation has emerged as a potentially relevant translational strategy for benign breast lesions, with previous studies reporting regression in approximately 70% of selected benign breast cases (1). However, previous studies lacked a standardized outpatient workflow capable of integrating dosage definition, operational reproducibility, short-interval monitoring, and response-oriented clinical decision support. Iodine is selectively absorbed by mammary epithelial cells via sodium-iodide symporters and has been associated with redox regulation, apoptotic signaling, endocrine modulation, and architectural reorganization in benign nodular conditions (7–10, 27–29). The systemic relationship between iodine intake and cancer risk remains complex and context-dependent. Zimmermann and Galetti (2015) (30), in a comprehensive review of animal and human studies, highlighted that both iodine deficiency and chronic excess can influence carcinogenic processes through mechanisms involving thyroidal and extrathyroidal oxidative stress, hormonal modulation, and epithelial proliferation.

To optimize delivery feasibility, outpatient usability, and local bioactivity, the protocol was developed under the principles of Evidence-Informed Design (EID) (31, 32), integrating mechanistic plausibility with operational and safety constraints to generate a reproducible outpatient intervention model. One of the objectives was to select and validate vehicles capable of facilitating intracellular delivery of bioactive agents and modulating oxidative, inflammatory, and perfusion-related dynamics in mammary and oncologic models.

Given this context, the present study describes the development and exploratory outpatient implementation of a scalable physiology-guided protocol for BI-RADS 2–4B breast nodules. The study integrates distinct but sequential methodological components comprising systematic evidence synthesis, translational prioritization, protocol development, and exploratory clinical implementation. These components were designed to support short-interval physiological modulation and response-oriented functional assessment while preserving established biopsy-based diagnostic pathways and oncologic vigilance. By integrating mechanistic evidence with operational feasibility and imaging-based decision support, the proposed framework seeks to transform dispersed physiological knowledge into a testable outpatient care pathway aligned with value-based healthcare principles and resource-sensitive clinical settings.

## 2 Methods

This study was conducted as a translational research project structured around complementary conceptual and operational methodological layers designed to integrate mechanistic evidence synthesis with outpatient protocol implementation. The methodological strategy was grounded in the principles of Evidence-Informed Design (EID) (31, 32), which support the integration of scientific evidence, clinical feasibility, stakeholder-oriented usability, and translational applicability within healthcare innovation processes.

Building upon methodological trajectories previously applied in translational and healthcare-innovation studies (33–37), the present study adopted a structured translational workflow designed to integrate mechanistic evidence synthesis with outpatient protocol implementation.

The methodological architecture combined distinct methods according to their specific function within the translational workflow. Work Breakdown Structure (WBS) methodology (38, 39) provided the overarching organizational structure through six sequential and interconnected Work Packages (WPs). Within this structure, systematic evidence synthesis was conducted in WP2 and WP4 according to PRISMA 2020 reporting principles (40); multicriteria decision analysis (MCDA) (41) supported translational prioritization in WP3 and WP5; and Design Thinking principles (Brown, 2008) (42) and Open Innovation strategies (Chesbrough, 2003) (43) supported protocol development and implementation-oriented translation. WP6 constituted a separate exploratory clinical implementation stage designed to assess imaging-response patterns, tolerability, operational feasibility, and compatibility with established diagnostic pathways rather than therapeutic efficacy.

**Figure 1:**
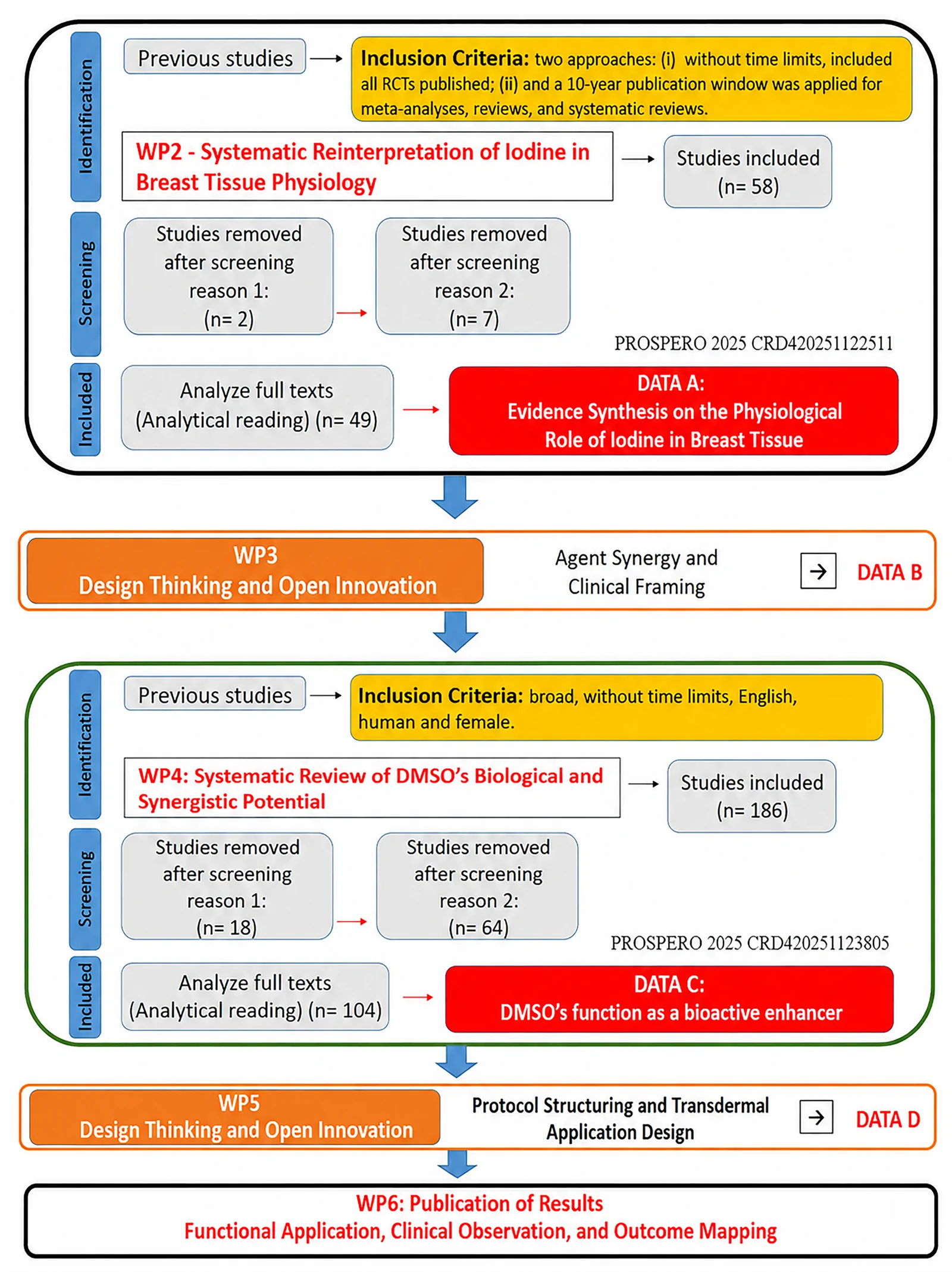
Structured Methodological Framework for Translational Development of a Physiological Modulation Protocol in Breast Nodule Management.

Note. Figure 1 illustrates the structured translational workflow adopted in this study, integrating systematic evidence synthesis, mechanistic prioritization, protocol structuring, and exploratory outpatient implementation across the six interconnected Work Packages (WPs).

Accordingly, the six WPs represent sequential but methodologically distinct components of a single translational development process: evidence synthesis (WP2 and WP4), translational prioritization and clinical framing (WP3), protocol structuring and delivery-route selection (WP5), and exploratory clinical implementation (WP6), under the governance structure established in WP1. This distinction was maintained throughout analysis and interpretation so that evidence derived from systematic synthesis, methodological prioritization, and exploratory clinical observations was not treated as representing the same level of evidence. Figure 1 summarizes this methodological architecture and the relationship among its components.

### 2.1 WP1—strategic project governance and open innovation integration

WP1 established the coordination and methodological organization of the research process using an Open Innovation-oriented logic42. A multidisciplinary team defined a governance structure focused on translational applicability, outpatient feasibility, and implementation reproducibility. Clinical workflows, imaging accessibility, monitoring routines, and operational constraints were mapped to ensure compatibility with low-complexity ambulatory settings and resource-sensitive clinical environments. Ethical boundaries for each stage were predefined, and all procedural documentation was centralized under internal research committee oversight.

Beyond operational coordination, WP1 functioned as the cross-cutting methodological governance layer of the study, ensuring that evidence synthesis, mechanistic plausibility, outpatient feasibility, and usability constraints remained coherently integrated across all subsequent Work Packages. Methodological quality control of the evidence-synthesis stages was coordinated within this governance structure and applied to WP2 and WP4. Given the heterogeneous translational evidence base, encompassing clinical, preclinical, *in vitro*, and mechanistic studies, evidence appraisal was conducted through sequential screening, full-text eligibility assessment, methodological and mechanistic relevance, consistency with the predefined objectives of each review, and suitability for translational synthesis. This structured appraisal supported analytical inclusion while preserving the distinct evidentiary contribution of different study designs. The corpus-specific screening, eligibility, and exclusion procedures are described in WP2 and WP4 and summarized in their respective PRISMA flow diagrams.

This governance structure enabled the progressive translation of physiological evidence into a structured outpatient intervention pathway while preserving methodological traceability, diagnostic safety, and alignment with conventional oncologic workflows. The overall methodological trajectory is illustrated in Figure 1, which summarizes the sequential integration of evidence synthesis, mechanistic prioritization, protocol structuring, and exploratory outpatient implementation; PRISMA 2020 reporting principles were specifically applied to the systematic evidence-synthesis components conducted in WP2 and WP4 (40).

### 2.2 WP2—systematic reinterpretation of iodine in breast-tissue physiology

WP2 consisted of a systematic review designed to synthesize and reinterpret evidence regarding the role of molecular iodine in breast tissue homeostasis, benign nodular modulation, and physiology-guided outpatient management strategies. Following PRISMA 2020 reporting principles (40), the review incorporated clinical, preclinical, and translational studies addressing iodine supplementation, redox modulation, mammary epithelial regulation, and microenvironmental signaling associated with benign breast lesions. The review protocol was prospectively registered in the PROSPERO database (44) under registration number CRD420251122511 and included both controlled clinical studies and mechanistic investigations relevant to translational implementation.

Although the original PROSPERO registration did not establish temporal restrictions, the evidence-selection strategy adopted in the present phase followed two complementary approaches to balance historical mechanistic consistency with contemporary translational applicability. The first approach, without publication-time limits, included all randomized controlled trials (RCTs) identified using the descriptors iodine, cancer, breast, humans, and female, yielding 26 eligible studies. In parallel, a 10-year publication window was applied to meta-analyses, reviews, and systematic reviews in order to prioritize clinically contemporary evidence reflecting advances in breast imaging, molecular diagnostics, and outpatient translational care. This second strategy identified 32 additional eligible studies.

**Figure 2:**
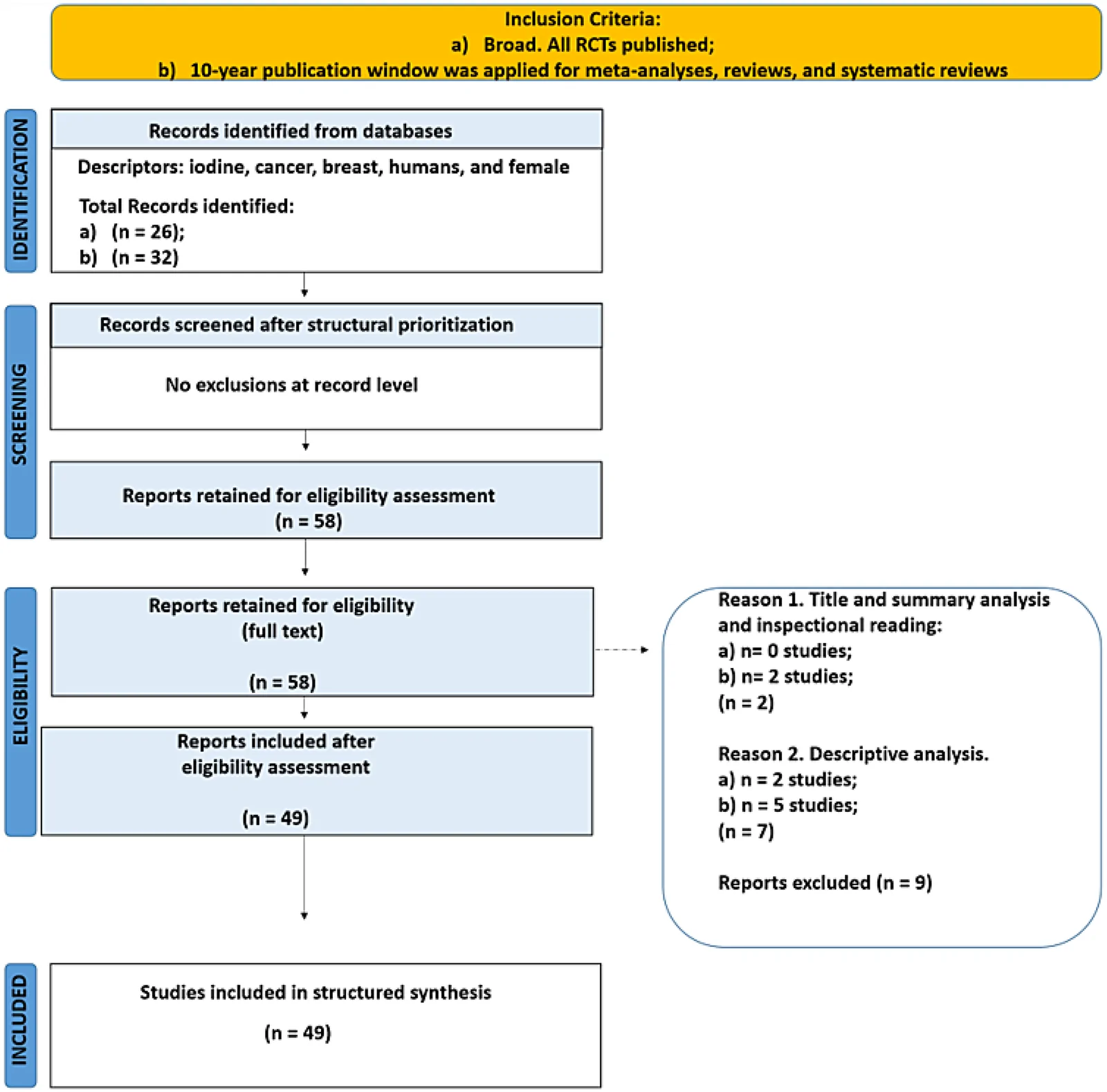
PRISMA flowchart for WP2 — systematic reinterpretation of iodine in breast tissue physiology.

Together, these complementary selection approaches resulted in a total of 58 studies included in the preliminary synthesis, of which 49 remained eligible for full-text analytical evaluation after screening exclusions. Study appraisal and analytical inclusion followed the methodological quality procedures established in WP1, with corpus-specific eligibility and exclusion decisions documented in the PRISMA flow diagram (Figure 2).

The inclusion of both mechanistic and higher-level evidence was maintained intentionally to support translational reinterpretation across molecular, physiological, and outpatient implementation domains. Rather than restricting the synthesis exclusively to conventional efficacy outcomes, WP2 sought to identify biologically coherent mechanisms potentially relevant to short-interval physiological modulation and response-oriented outpatient management.

Additional analytical refinement was incorporated during evidence synthesis to contextualize iodine-related mechanisms within broader microenvironmental domains, including redox balance, oxygen gradients, ionic transport regulation, and stromal signaling dynamics. Although exploratory in nature, this integrative perspective supported the subsequent identification of mechanistic domains potentially relevant to outpatient physiological modulation and functional imaging responsiveness in BI-RADS 2–4B lesions.

### 2.3 WP3—design thinking and open innovation for agent synergy and clinical framing

WP3 applied Design Thinking principles (42) integrated with Open Innovation strategies (43) to translate existing physiological and translational evidence into a feasible outpatient intervention model. This stage focused on identifying candidate agents capable of enhancing the biological and operational performance of iodine-based physiological modulation within BI-RADS 2–4B outpatient management.

The initial phase consisted of a scoping-style bibliographic search using predefined inclusion criteria, including English-language studies published within the previous 20 years and involving human, animal, or *in vitro* models relevant to iodine potentiation, transdermal delivery enhancement, redox modulation, or microenvironmental regulation. Searches were conducted in PubMed, Scopus, and Web of Science. Retrieved studies were screened for evidence supporting agents capable of improving dermal penetration, modulating oxidative and inflammatory dynamics, or synergizing with iodine-related biological effects in mammary tissue.

Relative weights were assigned according to the dual translational objective of preserving biological performance while maintaining operational simplicity and scalability. Mechanistic plausibility for synergistic interaction received the highest individual weight (40%), reflecting the requirement that candidate co-adjuvants demonstrate strong mechanistic alignment with the intended physiological modulation. Chemical compatibility and outpatient translational feasibility were each weighted at 30%, ensuring that biological potential remained balanced against formulation stability, safety, operational simplicity, and scalability within low-complexity ambulatory settings.

This prioritization process enabled the identification of candidate co-adjuvants capable of simultaneously supporting mechanistic coherence, outpatient usability, and operational scalability within the proposed physiology-guided intervention pathway.

### 2.4 WP4—systematic review of DMSO's biological and synergistic potential

Building upon the translational prioritization process established in WP3, dimethyl sulfoxide (DMSO) emerged as the most suitable candidate co-adjuvant for subsequent outpatient translational evaluation. WP4 therefore aimed to systematically assess its biological, physicochemical, and translational properties through structured evidence synthesis focused on its potential role as a bioactive enhancer within iodine-based physiological modulation strategies.

This stage consisted of a systematic review focused on the translational potential of DMSO in breast tissue modulation, particularly regarding its capacity to support dermal delivery, microenvironmental regulation, and functional synergy with molecular iodine. The review protocol was prospectively registered in the PROSPERO database (44) under registration number CRD420251123805 and conducted according to PRISMA 2020 reporting principles (40). Broad inclusion criteria were intentionally adopted—including no temporal restrictions, English-language publications, human studies, and female populations—to maximize mechanistic and translational evidence capture relevant to outpatient implementation.

**Figure 3:**
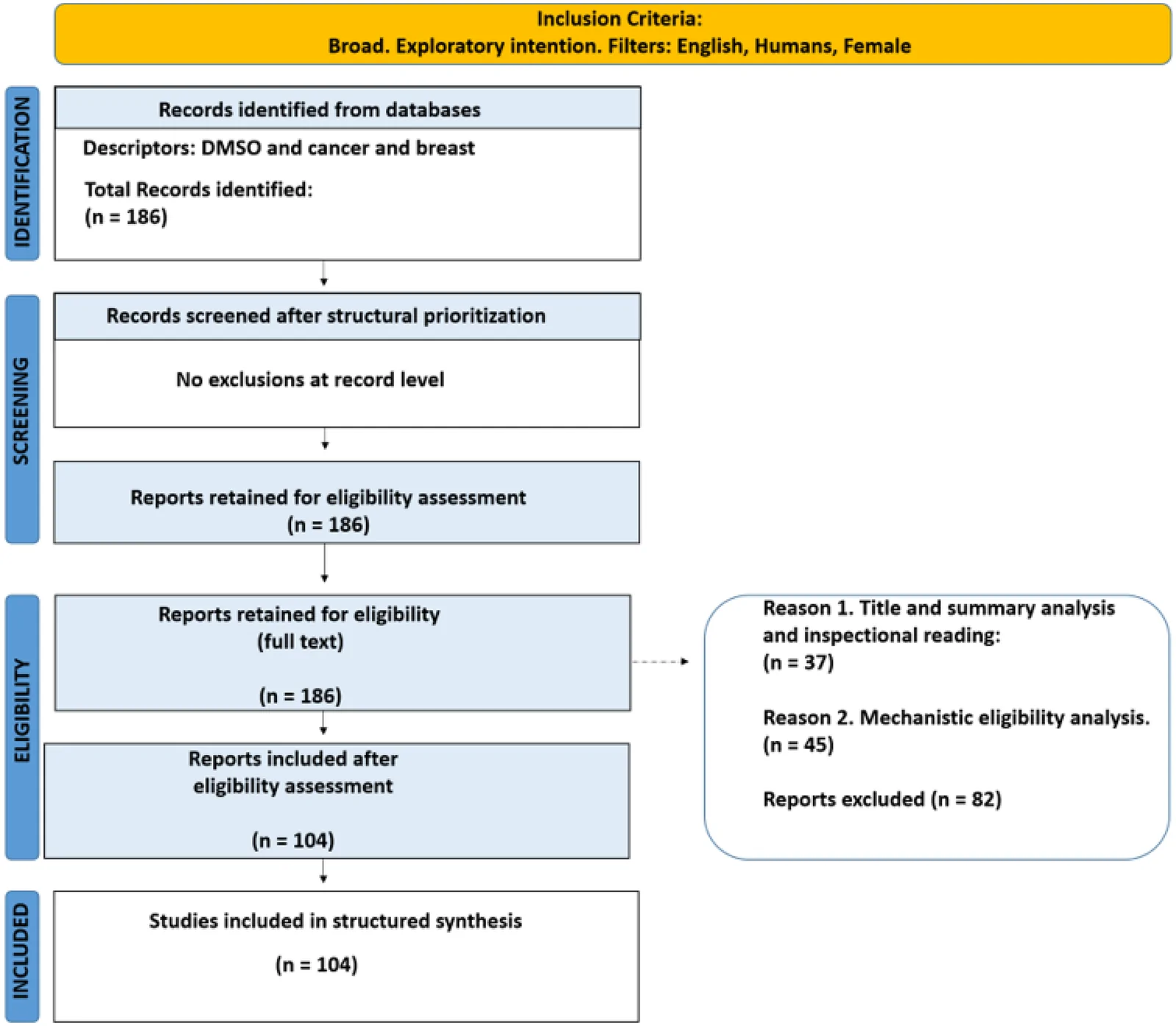
PRISMA flowchart for WP4 — systematic review on DMSO's biological and synergistic potential.

A total of 186 studies were identified through database searches, of which 104 remained eligible for full-text analytical evaluation after screening exclusions. Study appraisal and analytical inclusion followed the methodological quality procedures established in WP1, with corpus-specific eligibility and exclusion decisions documented in the PRISMA flow diagram (Figure 3).

The selected studies included *in vitro*, *in vivo*, and translational investigations addressing four mechanistic domains considered potentially relevant to outpatient physiological modulation: (i) redox modulation and oxidative-stress buffering, as central processes in tissue homeostasis and iodine-related antioxidant dynamics (45); (ii) transmembrane and dermal permeability enhancement, directly related to DMSO's capacity to facilitate molecular penetration and local bioavailability (46); (iii) epigenetic modulation, including histone deacetylase (HDAC)-related effects associated with chromatin remodeling and transcriptional regulation (47); and (iv) tissue-level immunomodulatory and perfusion-related mechanisms potentially associated with vascular normalization and microenvironmental responsiveness (48).

These mechanistic domains were prioritized due to their potential translational convergence with the biological and operational requirements identified in WP2, particularly regarding redox stability, tissue accessibility, microenvironmental modulation, and outpatient feasibility. Rather than functioning solely as a passive delivery vehicle, DMSO was investigated as a biologically active co-adjuvant potentially capable of amplifying tissue responsiveness, facilitating dermal penetration, and supporting the operational feasibility of short-interval physiology-guided modulation strategies.

The resulting evidence base provided the mechanistic and translational foundation necessary for subsequent outpatient protocol structuring in WP5 and exploratory clinical implementation in WP6.

### 2.5 WP5—protocol structuring and transdermal application design

WP5 focused on translating the mechanistic and translational evidence generated in previous Work Packages into a structured outpatient intervention model capable of real-world implementation in low-complexity clinical settings. Guided by Design Thinking principles (42) and Open Innovation strategies (43), this stage aimed to operationalize biological plausibility into a reproducible, usability-oriented protocol compatible with ambulatory workflows and short-interval physiological monitoring.

Candidate delivery routes—including oral, injectable, and transdermal formats—were comparatively evaluated through a multicriteria decision analysis (MCDA) framework (41, 49, 50). The evaluation matrix incorporated five predefined translational criteria: (i) compatibility with outpatient clinical workflows, (ii) mechanistic alignment with previously identified Critical Success Factors (CSFs), (iii) dosing predictability and reproducibility, (iv) patient tolerability and adherence potential, and (v) translational feasibility supported by existing literature (51).

Each candidate delivery format was comparatively scored on a direct-rating MCDA scale ranging from 0 to 2 (0 = not feasible; 1 = partially feasible; 2 = fully feasible). Each criterion was equally weighted in the composite score, reflecting the requirement that delivery-route selection simultaneously preserve clinical feasibility, mechanistic alignment, dosing predictability, tolerability and adherence, and translational applicability. This equal-weight structure was adopted to balance biological performance with operational simplicity and scalability without allowing any single dimension to dominate delivery-route selection.

This structured prioritization process enabled the identification of the delivery strategy most compatible with outpatient implementation, operational reproducibility, and physiology-guided modulation objectives. Particular emphasis was placed on balancing mechanistic coherence with feasibility, scalability, patient adherence, and implementation simplicity within resource-sensitive outpatient environments.

The finalized intervention model was subsequently described according to the Template for Intervention Description and Replication (TIDieR) checklist and guide (52) to ensure transparent reporting of materials, procedures, delivery format, application frequency, monitoring strategy, provider characteristics, implementation setting, and fidelity procedures. This reporting structure was adopted to strengthen reproducibility, implementation readiness, and translational applicability within outpatient clinical contexts.

### 2.6 WP6—exploratory clinical implementation and functional response evaluation

WP6 consisted of the exploratory outpatient implementation of the finalized iodine–DMSO protocol in three clinical cases involving BI-RADS 4B breast nodules, including two cases with concomitant contralateral BI-RADS 2 lesions. Post-intervention assessment incorporated ultrasound and magnetic resonance imaging (MRI) to evaluate functional responsiveness, imaging-response patterns, tolerability, and short-interval physiological modulation.

Although this exploratory implementation was not designed or powered to establish therapeutic efficacy, it was intended to examine whether the mechanistically derived protocol could generate clinically observable imaging-response patterns while simultaneously assessing feasibility, tolerability, and compatibility with established diagnostic escalation pathways. The observed responses were therefore interpreted as hypothesis-generating translational signals rather than confirmatory evidence of efficacy.

The implementation stage focused not only on preliminary clinical observation, but also on assessing the operational feasibility of the proposed outpatient workflow, including adherence, imaging integration, response-oriented monitoring, and compatibility with conventional diagnostic escalation pathways.

To strengthen translational consistency and implementation relevance, the protocol was additionally appraised using the SMART (53) and FINER (54) frameworks, supporting structured evaluation of specificity, feasibility, reproducibility, and clinical applicability within outpatient translational settings.

In addition to exploratory imaging outcomes, WP6 also incorporated safety assessment and translational outlook analysis, supporting a broader interpretation of outpatient physiology-guided modulation strategies and their potential role within response-oriented breast-nodule management pathways.

### 2.7 Ethics and consent

The study was conducted in accordance with the ethical principles of the Declaration of Helsinki (55), the International Council for Harmonisation—Good Clinical Practice guidelines (56), and applicable institutional requirements for clinical research governance and outpatient translational implementation.

All participants provided written informed consent prior to inclusion, authorizing both the clinical intervention and the use of anonymized imaging data for scientific dissemination and translational analysis. Given the exploratory and low-risk nature of the intervention—employing agents with established clinical safety profiles, including molecular iodine and dimethyl sulfoxide (DMSO)—the protocol underwent institutional ethical review and was approved by the internal research ethics committee of the coordinating institution in accordance with the International Ethical Guidelines for Health-related Research Involving Humans (57).

Patient confidentiality and imaging anonymization were maintained throughout all stages of outpatient implementation, analysis, and reporting, and no identifiable patient information is disclosed in this manuscript.

## 3 Results

The sequential integration of evidence across the methodological workflow provided progressive support for the proposed outpatient protocol, establishing its mechanistic plausibility, translational coherence, and implementation feasibility. Each analytical stage contributed specific outputs that collectively informed the design, prioritization, and operational structuring of the physiology-guided intervention pathway proposed for BI-RADS 2–4B breast nodules.

The following sections summarize the principal findings derived from the systematic reinterpretation of iodine physiology, the structured identification of translational mechanisms associated with tissue responsiveness, and the mechanistic integration of dimethyl sulfoxide (DMSO) as a biologically active co-adjuvant within the proposed outpatient modulation strategy. Together, these findings established the cross-systematic translational plausibility supporting the subsequent operational structuring and exploratory outpatient implementation of the protocol.

### 3.1 Translational mechanisms associated with iodine-responsive physiological modulation

The WP2 systematic reinterpretation of the physiological, biochemical, and translational evidence surrounding molecular iodine (I₂) enabled the identification of key biological domains associated with breast tissue homeostasis and benign nodular modulation. Building upon the early mechanistic hypothesis proposed by Clur (2003) (58) and the physiological framework later consolidated by Aceves et al. (2005) (59), the analysis integrated mechanistic, preclinical, and clinical evidence to identify convergent pathways related to redox balance, apoptosis, endocrine modulation, immune signaling, vascular responsiveness, and microenvironmental regulation.

To organize these convergent mechanisms within a translational outpatient perspective, the synthesized evidence was structured into two complementary analytical layers using a multicriteria decision analysis (MCDA) logic (41). The first layer comprised the principal translational outcome domains associated with iodine exposure, including apoptotic induction, endocrine modulation, epigenetic regulation, and immune potentiation. The second layer comprised the mechanistic and operational conditions required for these physiological effects to emerge, including redox stability, tissue accessibility, vascular responsiveness, ionic transport regulation, and microenvironmental permissiveness.

Table 1 summarizes the principal translational outcome domains associated with iodine-responsive physiological modulation, while Table 2 presents the corresponding mechanistic conditions identified as necessary for their functional expression. Together, these constructs established the biological and translational foundation supporting subsequent mechanistic prioritization, synergistic integration, and outpatient protocol development.

**Table 1:** Principal translational outcome domains (FPVs) associated with iodine-responsive physiological modulation.

FPV	Description	Supporting References
Apoptotic induction	Selective activation of programmed cell-death pathways associated with reduction of proliferative potential and physiological regression of nodular tissue.	Cuenca-Micó & Aceves (2020) (60); Cuenca-Micó et al. (2021) (61); Moreno-Vega et al. (2019) (62); Peña et al. (2020) (63).
Epigenetic modulation	Regulation of gene-expression patterns through methylation, chromatin remodeling, and transcriptional modulation favoring tumor-suppressive biological profiles.	Cuenca-Micó et al. (2021) (61); Moreno-Vega et al. (2019) (62).
Endocrine modulation	Modulation of hormone-related signaling pathways and receptor responsiveness associated with alterations in breast-tissue biological behavior.	Smyth (2016) (64); Dong et al. (2018) (65); Nappi et al. (2022) (66); Reiners et al. (2020) (67); Shim et al. (2021) (68); Zbigniew (2017) (69); Kryczyk-Kozioł et al. (2025) (70).
Immune potentiation	Activation and modulation of innate and adaptive immune-response pathways associated with enhanced tissue surveillance and tumor-recognition potential.	Cuenca-Micó & Aceves (2020) (60); Cuenca-Micó et al. (2021) (61); Moreno-Vega et al. (2019) (62).

**Table 2:** Mechanistic conditions (CSFs) associated with iodine-responsive physiological modulation.

CSF	Description	Supporting References
Redox modulation and oxidative-stress balance	Regulation of the tumor microenvironment redox state toward pro-apoptotic, anti-proliferative, and physiologically stabilizing conditions.	Cuenca-Micó & Aceves (2020) (60); Zbigniew (2017) (69); Ben-Yehuda Greenwald et al. (2017) (71); Cuellar-Rufino et al. (2017) (72); Cazarin et al. (2022) (73).
Immune activation and inflammatory polarization	Polarization of immune-response pathways (Th1/Th17 + IFN-*γ*↑/TGF-β↓) toward pro-inflammatory and antitumor biological profiles associated with enhanced tissue surveillance and tumor suppression.	Cuenca-Micó et al. (2021) (61); Moreno-Vega et al. (2019) (62).
Sodium-iodide symporter (NIS) expression and functional regulation	Increased NIS expression and membrane functionality enabling iodine uptake and responsiveness, with endocrine regulation involving estradiol, prolactin, and oxytocin signaling pathways.	De la Vieja & Santisteban (2018) (10); Cazarin et al. (2022) (73); Kelkar et al. (2016) (74); Kelkar et al. (2016) (2017) (75); Diocou et al. (2017) (76); Fletcher et al. (2020) (77).
Estrogen-receptor iodination	Post-translational iodination of estrogen-receptor tyrosyl residues associated with altered receptor affinity and downstream endocrine signaling in iodine-responsive breast tissue.	De la Vieja & Santisteban (2018) (10); Winder et al. (2022) (29).
Vascular modulation and perfusion responsiveness	Alterations in microcirculatory dynamics potentially improving iodine delivery, tissue distribution, and physiological responsiveness within breast tissue.	Coffey & Jochelson (2022) (78); Pötsch et al. (2022) (79); Shahraki et al. (2022) (80); Kornecki (2022) (81).
Microenvironmental modulation of ionic transport and bioenergetic gradients	Influence of extracellular pH, oxygen availability, redox balance, sodium-gradient stability, stromal signaling, and tissue-level physiological dynamics on ionic trafficking, membrane localization, and intracellular retention of transport-related mechanisms.	Gaspary et al. (2024) (33); Edgar et al. (2025) (36); Ben-Yehuda Greenwald et al. (2017) (71); Schug et al. (2018) (82); Shiozaki et al. (2019) (83); Skourti et al. (2023) (84); Volpe et al. (2020) (85).

Beyond the identification of broader translational outcome domains and mechanistic conditions, the evidence synthesis also revealed several biologically observable indicators potentially associated with tissue-level responsiveness to iodine-related modulation. Elevated sodium-iodide symporter (NIS) expression and membrane localization, preservation of redox-sensitive signaling pathways, maintenance of endocrine-regulatory axes, and imaging-detectable iodide uptake patterns emerged as recurrent mechanistic indicators associated with functional iodine responsiveness in mammary tissue (74–77, 86).

Collectively, these observations reinforce the translational relevance of the mechanistic conditions identified in the evidence synthesis by linking intracellular regulatory dynamics to measurable tissue-level and imaging-observable physiological responses. This integrative perspective further supported the subsequent prioritization of short-interval functional responsiveness as a relevant outpatient translational endpoint within the proposed physiology-guided intervention pathway.

**Figure 4:**
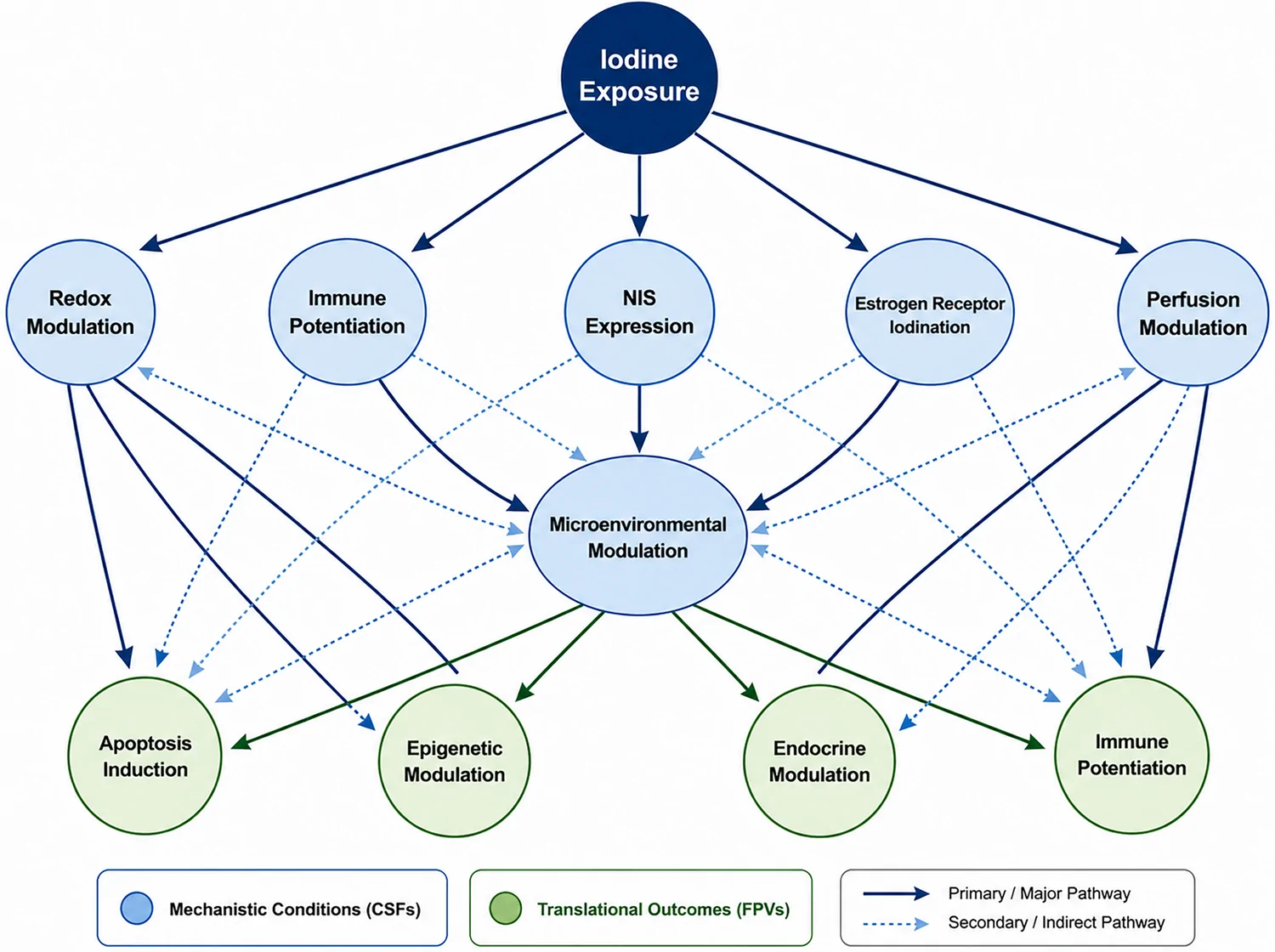
Mechanistic integration diagram linking critical success factors (CSFs) to fundamental points of view (FPVs) in iodine-responsive breast tissue.

Figure 4 illustrates the causal architecture derived from this synthesis, demonstrating how the identified mechanistic conditions interact to support broader physiological and translational response patterns within iodine-responsive breast tissue.

### 3.2 Translational convergence and prioritization of mechanisms associated with iodine-responsive modulation

Building upon the translational outcome domains and mechanistic conditions identified in the evidence synthesis, the subsequent WP3 analytical stage focused on understanding how these mechanisms converge to generate physiologically observable and clinically relevant response patterns within BI-RADS 2–4B breast nodules.

Apoptotic induction emerged as one of the principal translational response domains associated with iodine exposure and was supported by a convergent interaction among redox regulation, epigenetic modulation, and vascular responsiveness. Restoration of oxidative balance favoring pro-apoptotic signaling through reactive oxygen species (ROS) modulation (60, 69, 71–73), combined with chromatin remodeling and cytokine-related transcriptional reprogramming (61, 62), was consistently associated with tissue-level physiological changes potentially compatible with reduced lesion conspicuity and functional modulation. Complementary vascular and perfusion-related mechanisms (78–81) further supported the biological plausibility of iodine delivery within responsive tissue microenvironments. Clinical studies evaluating molecular iodine in conjunction with conventional therapeutic approaches also reported biological-response patterns consistent with apoptosis-associated signaling activation (87–89).

Epigenetic modulation similarly emerged as a stable physiological response domain associated with redox-sensitive chromatin regulation and immune-related transcriptional remodeling (61, 62). These mechanisms were linked to broader microenvironmental shifts potentially associated with tissue stabilization, reduced proliferative behavior, and altered biological responsiveness within iodine-exposed tissue niches (59, 61).

Immune-related physiological modulation was associated with Th1- and Th17-related signaling pathways characterized by increased IFN-*γ* expression, suppression of TGF-*β* activity, and enhanced cytotoxic immune responsiveness (61, 62). Importantly, these immune-related mechanisms appeared functionally interconnected with redox stabilization and vascular responsiveness, suggesting that iodine-mediated physiological modulation may depend on broader microenvironmental permissiveness rather than isolated intracellular signaling alone (87, 88).

Across these translational response domains, vascular modulation and microenvironmental regulation emerged as central integrative mechanisms connecting molecular activation to clinically observable tissue-level responses. Perfusion-related imaging approaches, including contrast-enhanced breast imaging techniques84–87, demonstrated particular translational relevance because they provide dynamic functional information potentially associated with tissue responsiveness, physiological modulation, and short-interval biological change detection. Likewise, microenvironmental regulation involving oxygen availability, ionic stability, stromal signaling, and redox balance (33, 36, 71, 82–84) emerged as a recurrent determinant of tissue-level responsiveness within iodine-responsive physiological contexts.

To comparatively prioritize mechanisms with greater translational applicability for subsequent outpatient implementation, the synthesized evidence was additionally evaluated through a multicriteria decision analysis (MCDA) logic (41) incorporating four translational dimensions: (i) projected clinical relevance for BI-RADS 2–4B outpatient management, (ii) consistency of mechanistic evidence across the 2015–2025 corpus, (iii) feasibility of short-interval outpatient monitoring, and (iv) operational safety within ambulatory settings. This structured prioritization process highlighted the redox–immune axis, vascular responsiveness, and tissue-accessibility mechanisms as central determinants of physiology-guided modulation strategies.

Taken together, these convergent findings suggest that iodine-related physiological modulation may operate as an active outpatient translational strategy capable of inducing measurable tissue-level responses rather than functioning exclusively as passive supplementation during surveillance. At the same time, the synthesis also identified important constraints—including tissue accessibility, perfusion heterogeneity, and microenvironmental instability—that could potentially limit uniform responsiveness across BI-RADS 2–4B lesions.

These mechanistic and operational limitations supported the subsequent investigation of catalytic co-adjuvants capable of enhancing dermal penetration, stabilizing tissue-level responsiveness, and improving outpatient implementation feasibility. To comparatively evaluate candidate synergistic agents, a structured translational prioritization matrix was developed incorporating three predefined criteria: chemical compatibility with iodine, mechanistic plausibility for synergistic interaction, and outpatient translational feasibility.

Table 3 summarizes the comparative translational prioritization process applied to candidate co-adjuvants. Each candidate was comparatively scored using a direct-rating MCDA scale ranging from 0 to 2 (0 = not feasible; 1 = partially feasible; 2 = fully feasible), with relative weights of 30% for chemical compatibility, 40% for mechanistic plausibility, and 30% for outpatient translational feasibility, as defined in WP3. Recent chemical and translational studies investigating iodine–DMSO interactions (Jayram & Jeena, 2018; Naidoo & Jeena, 2019; Monga et al., 2018; Li et al., 2020) (90–93) further supported the prioritization process.

**Table 3:** Translational prioritization matrix for candidate Co-adjuvants associated with iodine-responsive outpatient modulation.

Translational Criterion	Description	Scoring Scale	Relative Weight	Outcome Associated with DMSO
Chemical compatibility with iodine	Assessment of physicochemical stability, catalytic compatibility, and redox neutrality when combined with molecular iodine under physiological conditions.	0–2	30%	The DMSO/I₂ system demonstrated physicochemical stability and catalytic redox cycling properties compatible with safe topical application and reversible iodine regeneration.
Mechanistic plausibility for synergistic interaction	Evidence supporting enhancement of iodine-related physiological effects, including redox modulation, tissue accessibility, immune-related responsiveness, and apoptosis-associated signaling.	0–2	40%	DMSO demonstrated complementary antioxidant, permeability-enhancing, and microenvironmental-stabilizing properties supported by preclinical and translational evidence.
Translational feasibility for outpatient implementation	Suitability for ambulatory application considering safety, dosing practicality, operational reproducibility, and compatibility with outpatient workflows.	0–2	30%	DMSO demonstrated high outpatient applicability due to established human use, low toxicity profile, operational simplicity, and compatibility with transdermal administration.

*Translational prioritization parameters informed by Jayram & Jeena (2018) (90); Naidoo & Jeena (2019) (91); Monga et al. (2018) (92); Li et al. (2020) (93).

The resulting prioritization process identified dimethyl sulfoxide (DMSO) as the most translationally compatible candidate co-adjuvant for subsequent mechanistic and outpatient evaluation. Experimental evidence demonstrated that the DMSO/I₂ system exhibits physicochemical stability and catalytic redox cycling properties capable of maintaining iodine bioavailability without oxidative degradation of either component (90–93). These characteristics supported the biological plausibility, operational safety, and outpatient feasibility required for subsequent physiology-guided implementation.

### 3.3 DMSO as a translational co-adjuvant in iodine-responsive modulation

The structured evidence synthesis investigating dimethyl sulfoxide (DMSO) demonstrated that several of its biological and physicochemical properties converge with mechanistic domains previously associated with iodine-responsive physiological modulation in breast tissue. This analytical stage therefore focused on determining whether DMSO-related mechanisms could reinforce tissue responsiveness, improve outpatient feasibility, and support physiological modulation within BI-RADS 2–4B nodular contexts.

Table 4 summarizes the principal WP2 translational response domains potentially reinforced by DMSO co-intervention. Among these, apoptosis-associated modulation and epigenetic regulation emerged as the most consistently supported biological domains identified through the systematic evidence synthesis.

**Table 4:** WP2 translational response domains (FPVs) potentially reinforced by DMSO Co-intervention.

WP2 FPV	Description	Supporting References
Apoptosis-associated modulation	DMSO has been associated with differentiation-related reduction of proliferative potential and partial reversal of malignant cellular phenotypes. When combined with cytotoxic or redox-active interventions, these mechanisms may reinforce iodine-related apoptosis-associated physiological modulation.	Friend et al. (1971) (94); Collins et al. (1978) (95); Caron et al. (2010) (96); Ross (1985) (97); Scher et al. (1982) (98).
Epigenetic modulation	DMSO-related differentiation processes have been associated with modulation of gene expression, membrane glycoproteins, and transcription-related cellular patterns potentially compatible with broader epigenetic responsiveness.	Brown et al. (1982) (99); Gahmberg et al. (1979) (100); Tarella et al. (1982) (101); Higgins & Borenfreund, (1980) (102).

Importantly, mechanistic domains such as endocrine modulation and immune potentiation were not directly attributed to DMSO because the available evidence does not support these pathways as primary independent biological response domains for DMSO exposure. Although DMSO may facilitate steroid penetration (103) and influence membrane-associated transport dynamics without demonstrating direct endocrine-regulatory activity comparable to iodine (104), its reported anti-inflammatory, lysosome-stabilizing, and membrane-protective effects (105–107) appear to function primarily as permissive microenvironmental mechanisms rather than as direct endocrine or immune-response activators.

The synthesis further demonstrated that DMSO-related biological effects predominantly converge at the level of tissue accessibility, redox buffering, perfusion responsiveness, and membrane-level physiological modulation. Table 5 summarizes the principal WP2 mechanistic conditions potentially supported by DMSO co-intervention.

**Table 5:** WP2 mechanistic conditions (CSFs) potentially supported by DMSO Co-intervention.

WP2 CSF	Description	Supporting References
Redox modulation and oxidative-stress buffering	DMSO acts as a scavenger of reactive oxygen species and supports stabilization of oxidative conditions within ischemic and inflamed tissues without completely suppressing apoptosis-associated signaling.	Sanmartín-Suárez et al. (2011) (108); Ashwood-Smith (1975) (109); Del Maestro et al. (1980) (110); Ghosh et al. (1976) (111); Schlafer et al. (1982) (112); Panganamala et al. (1976) (113).
Vascular modulation and perfusion responsiveness	DMSO-related effects on platelet aggregation, endothelial integrity, and microvascular stability may improve tissue perfusion and physiological distribution of iodine within heterogeneous nodular environments.	Jacob & Herschler (1986 (114); Dujovny et al. (1983) (115); Finney et al. (1967) (116); Rosenblum & El-Sabban (1982) (117); Sullivan & Gad (2024) (118).
Microenvironmental modulation and tissue accessibility	DMSO enhances dermal and membrane permeability through biophysical modulation of lipid organization and membrane dynamics, facilitating tissue penetration, diffusion, and local physiological accessibility.	Maibach & Feldmann (1967) (103); Kligman (1965) (119); Turco & Canada (1969) (120); Gordeliy et al. (1998) (121); Kennedy et al. (2003) (122); Schrader et al. (2016) (123); Gironi et al. (2020) (124).

Mechanistic pathways directly dependent on iodine-specific biology—such as sodium-iodide symporter (NIS) regulation and estrogen-receptor iodination—were not attributed to DMSO because no available evidence demonstrated that DMSO independently induces these molecular processes (10, 73–77). Instead, DMSO-related effects appear predominantly associated with membrane-level, microenvironmental, and physicochemical facilitation mechanisms (114, 124).

Beyond these mechanistic conditions, the synthesis also identified broader translational contributions potentially associated with DMSO co-intervention, including enhancement of apoptosis-associated selectivity through controlled oxidative buffering (117, 124, 125), improved tolerability through inflammatory-noise reduction (105–108), facilitation of tissue penetration in regions with limited NIS functionality (103, 114), and support of physiological distribution across heterogeneous tissue microenvironments (114, 116, 117, 124, 126, 127). Table 6 summarizes the specific translational contributions associated with DMSO co-intervention and their potential outpatient implications.

**Table 6:** Specific translational contributions of DMSO within iodine-responsive physiological modulation.

Translational Domain	Baseline Iodine-Related Mechanism	Potential Contribution of DMSO	Potential Outpatient Implication
Apoptosis-associated modulation	Pro-apoptotic physiological modulation associated with iodine-related oxidative signaling94	Controlled oxidative buffering potentially preserving selective physiological responsiveness while reducing excessive oxidative injury (117, 124, 125).	Improved tissue tolerability and stabilization of apoptosis-associated physiological modulation
Tolerability and inflammatory buffering	Immune-related physiological modulation associated with iodine exposure67	Anti-inflammatory and membrane-stabilizing effects potentially reducing excessive inflammatory activation (105–108).	Improved tolerability and maintenance of outpatient adherence
Tissue accessibility in low-responsiveness regions	Iodine uptake dependent on NIS-related transport and passive diffusion10	Enhancement of dermal and interstitial penetration within poorly perfused or fibrotic tissue regions (103, 114).	Increased local accessibility and physiological responsiveness
Perfusion-related physiological distribution	Perfusion-dependent iodine distribution within nodular tissue84	Improvement of microvascular stability and physicochemical diffusion across heterogeneous tissue environments (114, 116, 117, 124, 126, 127).	Improved tissue distribution and short-interval physiological accessibility

Collectively, these findings support the interpretation of DMSO not merely as a dermal carrier, but as a biologically active co-adjuvant capable of enhancing tissue accessibility, stabilizing microenvironmental responsiveness, and improving outpatient implementation feasibility within iodine-responsive physiological modulation strategies. These convergent properties provided the translational rationale supporting subsequent protocol structuring and exploratory outpatient implementation.

## 4 Protocol design and outpatient structuring

Building upon the mechanistic convergence and translational prioritization processes described in the previous sections, the subsequent analytical stage focused on converting physiological plausibility into a structured outpatient intervention model compatible with real-world clinical implementation. This stage integrated translational feasibility, outpatient usability, delivery-route prioritization, and operational reproducibility to define a physiology-guided protocol suitable for BI-RADS 2–4B breast-nodule management.

Particular emphasis was placed on identifying delivery strategies capable of balancing mechanistic coherence, tissue accessibility, patient tolerability, workflow integration, and short-interval monitoring feasibility within low-complexity ambulatory settings. The resulting protocol architecture sought to preserve alignment with conventional oncologic pathways while enabling response-oriented physiological modulation and outpatient functional assessment.

The following sections describe the comparative prioritization of candidate delivery routes, the operational structuring of the transdermal protocol, and the implementation logic supporting reproducibility, monitoring fidelity, and translational applicability within outpatient clinical environments.

### 4.1 Translational prioritization of the delivery strategy

WP5 focused on translating the mechanistic synthesis generated in previous analytical stages into an operational outpatient intervention model through the comparative prioritization of candidate delivery routes. Building upon the mechanistic convergence and translational plausibility established across previous sections, oral, injectable, and transdermal formats were comparatively assessed through a multicriteria decision analysis (MCDA) logic (41, 49, 50, 158) integrating mechanistic coherence, operational feasibility, dosing reproducibility, patient tolerability, and translational applicability.

Five predefined translational criteria guided the comparative assessment: (i) compatibility with outpatient clinical workflows, (ii) mechanistic alignment with previously identified physiological-response mechanisms, (iii) dosing predictability and reproducibility, (iv) patient tolerability and adherence potential, and (v) feasibility of implementation within low-complexity ambulatory settings (51).

The transdermal configuration achieved the highest composite score (10/10), outperforming oral administration—which demonstrated limitations related to systemic variability and reduced dosing predictability—and injectable approaches, which were limited by invasiveness, workflow complexity, and lower outpatient applicability. Table 7 summarizes the comparative translational prioritization applied to the candidate delivery routes.

**Table 7:** Comparative translational prioritization of candidate delivery routes.

Delivery Route	Clinical Feasibility	Mechanistic Alignment	Dosing Predictability	Tolerability and Adherence	Translational Applicability	Total Score
Oral	2	1	0	1	1	5
Injectable	0	2	2	0	1	5
Transdermal	2	2	2	2	2	10

The prioritization of the transdermal route was further supported by mechanistic evidence demonstrating that iodine and iodide exposure can activate Nrf2-associated cytoprotective pathways in human skin, contributing to epithelial resilience and modulation of inflammatory signaling (71). These findings reinforced the biological plausibility of transdermal physiology-guided modulation within outpatient settings.

The resulting prioritization also aligned with contemporary evidence demonstrating that transdermal delivery systems constitute mature and operationally versatile platforms capable of improving tissue permeability, bypassing stratum corneum limitations, and supporting reproducible outpatient dosing strategies (126, 127). Together, these mechanistic and operational findings supported the adoption of a structured transdermal intervention model as the foundational outpatient configuration for subsequent protocol implementation.

### 4.2 Protocol design and operational structuring

Following the translational prioritization of the transdermal route, WP5 focused on converting the selected delivery configuration into a structured outpatient intervention model compatible with reproducible ambulatory implementation. The resulting protocol was designed to balance physiological plausibility, operational feasibility, tissue accessibility, patient tolerability, and monitoring reproducibility within low-complexity outpatient environments.

The operational structure of the intervention was informed by mechanistic evidence related to redox modulation, tissue permeability, and microenvironmental responsiveness, as well as by practical workflow considerations associated with outpatient delivery. Particular emphasis was placed on standardizing exposure conditions, dermal tolerability, formulation stability, anatomical targeting, and response-oriented monitoring strategies capable of supporting short-interval physiological assessment.

To ensure transparent reporting and implementation reproducibility, the finalized intervention model was structured according to the Template for Intervention Description and Replication (TIDieR) checklist and guide (52). This reporting framework incorporated the rationale, materials, procedures, provider characteristics, delivery format, application schedule, outpatient setting, and fidelity-monitoring procedures associated with the proposed protocol.

The resulting intervention model consisted of a structured iodine–DMSO transdermal protocol designed for outpatient management of BI-RADS 2–4B breast nodules through repeated short-interval physiological modulation sessions integrated with imaging-based functional assessment.

### 4.3 Intervention configuration and application procedure

The finalized outpatient intervention model consisted of a structured iodine–DMSO transdermal application protocol designed to maximize tissue contact, dermal tolerability, formulation stability, and operational reproducibility within ambulatory clinical settings. The intervention configuration incorporated physicochemical evidence related to iodine–DMSO compatibility (90–93), membrane permeability enhancement (103, 119–124), and tissue-level physiological accessibility associated with transdermal delivery systems (126, 127).

Each session utilized 2.5 mL of pharmaceutical-grade dimethyl sulfoxide (DMSO; 99.9%) applied onto a sterile 3 × 3 cm cotton pad that had been optionally pre-moistened with approximately 1 mL of isotonic saline solution (0.9% NaCl) in cases involving increased dermal sensitivity. Subsequently, 2.5 mL of Lugol's solution (5% iodine) was applied over the DMSO layer immediately before topical placement. This sequential configuration was adopted to minimize iodine volatilization and optimize tissue-level exposure.

The exposure regimen was pragmatically defined during protocol structuring to maximize local iodine exposure while preserving cutaneous tolerability and outpatient reproducibility. The selected volume and 10-minute exposure period were retained as a locally intensive configuration that remained compatible with cutaneous tolerability during exploratory implementation. Isotonic saline pre-moistening was incorporated solely as an operational tolerability adjustment for individuals presenting increased dermal sensitivity, particularly because repeated iodine exposure may induce mild transient irritation or superficial pigmentation changes; it was not intended to modify the iodine dose or the mechanistic rationale of the intervention.

Anatomical lesion targeting was performed using ultrasound or magnetic resonance imaging (MRI)-guided clock-face localization. Prior to application, the skin surface was cleansed using 70% alcohol to reduce contamination and avoid dilution-related interference with tissue exposure dynamics.

All applications were performed manually by trained outpatient clinical personnel using sterile disposable materials within ambulatory procedural environments. Protocol adherence, exposure time, and procedural fidelity were documented in real time during each session.

The intervention schedule consisted of ten outpatient sessions distributed over five weeks (two sessions weekly), with each session lasting exactly 10 min. Procedural fidelity was maintained through standardized operational checklists confirming skin preparation, exposure duration, residue removal, and monitoring documentation.

No serious adverse dermal reactions or protocol deviations were observed during outpatient implementation. Mild transient effects—including localized irritation or iodine-related pigmentation—were occasionally identified and adequately controlled through the saline pre-moistening strategy.

To preserve diagnostic safety and maintain compatibility with conventional oncologic workflows, a bilateral breast MRI checkpoint was incorporated after the sixth session (approximately week three) under institutional ethical oversight. This intermediate imaging assessment functioned as a response-oriented safety mechanism enabling early identification of limited responders without delaying conventional diagnostic escalation pathways. Cases maintaining BI-RADS 4 characteristics or imaging patterns requiring cytological clarification were referred for fine-needle aspiration (FNA) or additional evaluation according to NCCN Clinical Practice Guidelines in Oncology (128).

This operational structure allowed the protocol to function as a controlled outpatient translational bridge integrating short-interval physiological modulation with response-oriented imaging reassessment while preserving the integrity and timing of established oncologic management pathways. The standardized operational workflow, intervention components, procedural sequence, safety checkpoints, and implementation characteristics of the outpatient iodine–DMSO protocol are summarized in Table 8 following the TIDieR reporting framework.

**Table 8:** Standardized operational workflow of the iodine–DMSO outpatient protocol (TIDieR-aligned overview).

Step	Operational Description
1. Eligibility and baseline assessment	Identification of breast nodules within the proposed BI-RADS 2–4B translational scope, with baseline clinical and imaging assessment and confirmation that protocol implementation would not interfere with established diagnostic or oncologic management requirements.
2. Anatomical targeting	Anatomical lesion targeting using the clock-face localization method based on prior ultrasound or magnetic resonance imaging (MRI).
3. Skin preparation	Skin preparation with 70% alcohol followed by complete surface evaporation prior to topical exposure.
4. Intervention preparation	Preparation of a sterile 3 × 3 cm cotton pad containing 2.5 mL pharmaceutical-grade DMSO (99.9%) and 2.5 mL Lugol's solution (5% iodine); optional pre-moistening with approximately 1 mL isotonic saline solution (0.9% NaCl) in cases involving increased dermal sensitivity.
5. Transdermal application	Direct application over the targeted lesion for a standardized exposure duration of 10 min without occlusive covering, followed by pad removal and superficial cleaning of residual material without adjunct dressings or additional topical agents.
6. Intervention schedule	Two outpatient sessions weekly for five consecutive weeks, corresponding to a planned total of 10 sessions, subject to the predefined mid-protocol response and safety assessment.
7. Mid-protocol imaging checkpoint	Bilateral breast MRI after the sixth session (approximately week 3) to assess short-interval imaging responsiveness and preserve compatibility with conventional oncologic workflows.
8. Response-oriented decision and stopping rule	Cases demonstrating imaging normalization or a response pattern compatible with protocol continuation according to the predefined MRI and ultrasound parameters could proceed with the planned intervention. Persistence of BI-RADS 4 characteristics, limited imaging response, residual vascular signaling, or findings requiring cytological clarification triggered protocol discontinuation and referral for fine-needle aspiration (FNA) or additional diagnostic evaluation according to NCCN Clinical Practice Guidelines in Oncology (Gradishar et al., 2024) (128).

### 4.4 Operational replicability and translational implementation structure

One of the central translational characteristics of the present outpatient framework is its emphasis on operational reproducibility through the structured integration of mechanistic plausibility, implementation logic, response-oriented reassessment, and low-complexity outpatient feasibility. Rather than relying on isolated empirical experimentation, the protocol was progressively constructed through sequential translational stages integrating evidence synthesis, mechanistic prioritization, protocol structuring, and exploratory outpatient implementation.

This implementation architecture contributed directly to the reproducibility profile of the proposed intervention by linking biological rationale to operational decision-making. Mechanistic evidence synthesis supported physiological plausibility; multicriteria prioritization enabled structured translational decision support; outpatient protocol standardization facilitated reproducible implementation; and predefined imaging checkpoints preserved diagnostic compatibility with conventional oncologic workflows.

Importantly, the reproducibility of the present framework does not depend on specialized technological infrastructure or proprietary therapeutic systems. Instead, the intervention was designed around operational simplicity, standardized implementation sequences, predefined monitoring criteria, and response-oriented outpatient reassessment. These characteristics may facilitate future scalability and controlled translational validation across heterogeneous ambulatory environments.

Table 9 summarizes the principal operational components contributing to the translational reproducibility and outpatient implementation structure of the proposed iodine–DMSO protocol.

**Table 9:** Operational components supporting translational reproducibility and outpatient implementation.

Operational Component	Translational Contribution
Mechanistic evidence synthesis	Supported the biological plausibility of iodine-responsive physiological modulation pathways.
Multicriteria translational prioritization	Enabled structured selection of mechanistically compatible and operationally feasible outpatient strategies.
Transdermal delivery prioritization	Facilitated tissue accessibility, outpatient feasibility, and implementation reproducibility.
TIDieR-guided protocol structuring58	Standardized procedural reporting, intervention fidelity, and operational reproducibility.
Predefined MRI checkpoint134	Preserved diagnostic safety and compatibility with conventional oncologic escalation pathways.
Functional imaging responsiveness	Supported dynamic outpatient reassessment and response-oriented translational decision-making.
Low-complexity outpatient architecture	Facilitated scalability, operational simplicity, and compatibility with resource-sensitive clinical environments.

Taken together, these implementation characteristics suggest that the translational relevance of the present framework derives not only from the physiological effects associated with iodine–DMSO modulation, but also from the structured outpatient architecture through which these mechanisms were operationalized, monitored, and integrated into real-world ambulatory workflows. The subsequent section presents the exploratory clinical application of this protocol and the translational implications associated with functional imaging responsiveness, outpatient feasibility, and early physiology-guided decision-making.

## 5 Exploratory clinical implementation and translational applicability

Following the mechanistic prioritization and operational structuring stages developed in previous work packages, WP6 implemented the finalized iodine–DMSO transdermal protocol in an exploratory outpatient context to evaluate functional responsiveness, imaging-based physiological modulation, implementation feasibility, and compatibility with conventional oncologic workflows.

This implementation stage focused not only on preliminary clinical observation, but also on assessing whether short-interval physiology-guided modulation could generate measurable imaging-response patterns capable of supporting outpatient decision-making and response-oriented functional triage within BI-RADS 2–4B breast nodules.

Particular emphasis was placed on evaluating protocol tolerability, outpatient reproducibility, workflow integration, imaging responsiveness, and translational applicability under real-world ambulatory conditions. The following sections summarize the exploratory clinical observations, functional imaging outcomes, translational-response patterns, and implementation considerations derived from the outpatient application of the proposed intervention model.

### 5.1 Exploratory outpatient implementation and imaging-based functional responsiveness

WP6 implemented the finalized iodine–DMSO transdermal protocol in three exploratory outpatient cases involving BI-RADS 4B breast nodules, including two cases with concomitant contralateral BI-RADS 2 lesions. The implementation strategy incorporated a predefined bilateral breast magnetic resonance imaging (MRI) reassessment after the sixth session to preserve diagnostic safety and maintain compatibility with conventional oncologic workflows.

This intermediate imaging checkpoint functioned as a response-oriented operational safeguard, allowing early differentiation between physiologically responsive and limited-response patterns without delaying conventional diagnostic escalation. Cases demonstrating persistent BI-RADS 4 characteristics or imaging findings suggestive of continued cytological uncertainty were referred for fine-needle aspiration (FNA) or additional evaluation according to NCCN Clinical Practice Guidelines in Oncology (128).

The exploratory outpatient implementation revealed a graded physiological-response pattern across the three evaluated cases. Two cases (Cases 1 and 2) completed the ten-session protocol and demonstrated complete radiologic resolution of BI-RADS 4B nodules accompanied by normalization of contrast enhancement, vascular signals, and stromal tissue architecture. In both cases, concomitant contralateral BI-RADS 2 nodules also resolved within the same outpatient treatment interval. Case 1 (48 years old) maintained complete imaging remission during a 12-month follow-up period, while Case 2 (51 years old) demonstrated sustained structural remodeling during short-term surveillance.

In contrast, Case 3 (37 years old) demonstrated a partial imaging-response pattern after six sessions, with imaging downgrading from BI-RADS 4B to BI-RADS 4A. Following the predefined MRI checkpoint, the outpatient protocol was discontinued and the patient underwent fine-needle aspiration (FNA), which confirmed benign cytology. This controlled discontinuation illustrated the potential role of short-interval physiological responsiveness as an outpatient functional triage signal capable of guiding timely diagnostic escalation while avoiding unnecessary delays in conventional oncologic evaluation.

Collectively, these exploratory observations aligned with several mechanistic domains identified in previous analytical stages—including redox modulation, vascular responsiveness, tissue accessibility, and immune-related physiological modulation—and supported the interpretation of functional imaging responsiveness as a potentially relevant translational marker for short-interval outpatient decision-making in breast-lesion management. Table 10 summarizes the imaging outcomes, follow-up observations, and functional-response interpretations associated with the exploratory outpatient implementation cases.

**Table 10:** Imaging outcomes and functional-response patterns following exploratory outpatient implementation.

Case	Age	Baseline BI-RADS	Sessions	Post-Intervention MRI	Post-Intervention Ultrasound	Follow-up	Functional-Response Interpretation
1	48 y	4B/2	10	Complete Radiologic resolution	Normalized echogenicity with absence of Doppler vascular signal	NDC (12 mo)	Complete imaging-response pattern with contrast, vascular, and tissue-level normalization
2	51 y	4B/2	10	Complete Radiologic resolution	Structural softening and vascular-signal reduction	NDC (6 mo)	Sustained imaging-response pattern with structural remodeling and vascular-signal reduction
3	37 y	4B	6	Partial regression (BI-RADS 4A)	Partial regression with persistent Doppler vascular flow	FNA benign)	Limited-response pattern supporting functional triage and early diagnostic escalation

BI-RADS, Breast Imaging Reporting and Data System; NDC, no detectable change during surveillance imaging; FNA, fine-needle aspiration.

### 5.2 Functional responsiveness as a translational imaging marker

Within the exploratory outpatient implementation pathway, functional responsiveness emerged as a potentially relevant translational marker capable of integrating physiological modulation with imaging-based decision support. Rather than relying exclusively on static morphological classification, the proposed workflow incorporated short-interval dynamic imaging reassessment to evaluate whether tissue-level physiological changes could be detected during outpatient modulation.

Functional responsiveness was operationalized through predefined imaging-related parameters associated with tissue remodeling and physiological modulation. On magnetic resonance imaging (MRI), imaging response was characterized by complete or partial reduction of contrast-enhancement patterns accompanied by stromal normalization. On ultrasound, imaging response was characterized by reduction or extinction of Doppler vascular signals combined with echogenic remodeling. Complete imaging response required resolution of the target lesion together with normalization of the associated functional imaging abnormalities, whereas limited response was defined by persistence of residual lesion characteristics, vascular signaling, or imaging features requiring continued diagnostic clarification.

Across the exploratory implementation cases, functional responsiveness demonstrated two complementary translational implications. In Cases 1 and 2, pronounced responsiveness patterns were associated with imaging normalization and sustained structural remodeling, suggesting compatibility with physiological stabilization and outpatient conservative follow-up. In contrast, the limited-response pattern observed in Case 3 functioned as an early translational triage signal supporting interruption of the outpatient modulation phase and immediate referral for cytological clarification according to conventional oncologic workflows (128).

This response-oriented interpretation suggests that short-interval imaging reassessment may provide biologically informative signals extending beyond static lesion classification alone. Within this outpatient framework, functional responsiveness operated not merely as an imaging outcome, but as a dynamic translational indicator potentially capable of distinguishing adaptive physiological remodeling from persistent tissue-level alteration requiring diagnostic escalation.

Taken together, these findings support the interpretation that physiology-guided imaging responsiveness may contribute to outpatient decision-making by integrating tissue-level modulation patterns with conventional surveillance and biopsy pathways, thereby reinforcing the translational applicability of short-interval outpatient modulation strategies within BI-RADS 2–4B breast-nodule management.

### 5.3 Translational feasibility and structured outpatient applicability

Beyond the exploratory imaging outcomes, the outpatient implementation pathway was additionally evaluated regarding feasibility, operational reproducibility, translational applicability, and compatibility with low-complexity ambulatory settings. This assessment incorporated structured appraisal according to the SMART (53) and FINER (54) frameworks to examine the protocol's translational robustness and implementation coherence.

The proposed intervention fulfilled all SMART criteria (53). The protocol was specific in targeting BI-RADS 2–4B breast nodules through a standardized transdermal outpatient regimen; measurable through MRI- and ultrasound-based responsiveness parameters; achievable within ambulatory settings with complete protocol adherence; relevant to the unmet need for active outpatient management of indeterminate breast nodules; and time-bound through a predefined five-week intervention cycle with short-interval imaging reassessment.

Likewise, the intervention fulfilled the FINER criteria (54) by demonstrating feasibility within low-complexity outpatient environments, translational interest as a physiology-guided outpatient modulation strategy, ethical compatibility under institutional oversight, and potential scientific and clinical relevance for response-oriented breast-nodule management. Table 11 summarizes the structured translational appraisal of the iodine–DMSO outpatient implementation model according to SMART and FINER criteria.

**Table 11:** SMART and FINER appraisal of the iodine–DMSO outpatient protocol.

Criterion	Translational Appraisal
Specific	Standardized outpatient intervention targeting BI-RADS 2–4B breast nodules through a defined transdermal workflow.
Measurable	MRI and ultrasound responsiveness parameters including contrast-enhancement reduction, Doppler-signal modulation, and structural remodeling.
Achievable	Full outpatient adherence to the predefined workflow, with protocol completion or imaging-guided discontinuation according to established reassessment criteria.
Relevant	Addresses the translational gap between passive surveillance and invasive escalation in low- and intermediate-suspicion breast nodules.
Time-bound	Structured five-week intervention schedule incorporating predefined short-interval imaging reassessment.
Feasible	Operationally compatible with low-complexity ambulatory implementation and minimal procedural infrastructure.
Interesting	Introduces response-oriented physiological modulation as a complement to conventional surveillance workflows.
Novel	First structured iodine–DMSO transdermal outpatient implementation model incorporating short-interval imaging-based functional reassessment.
Ethical	Non-invasive outpatient intervention conducted under informed consent and institutional ethical oversight.
Relevant (scientific and clinical)	Supports translational integration between physiological modulation, outpatient decision-making, and response-oriented imaging assessment.

Collectively, these findings reinforce the translational applicability of the proposed outpatient workflow by demonstrating that the iodine–DMSO protocol can be operationalized through a reproducible, ethically compatible, and implementation-oriented framework suitable for exploratory physiology-guided outpatient management.

### 5.4 Safety considerations and operational safeguards

Despite the favorable exploratory outpatient outcomes, several safety domains required explicit consideration to preserve compatibility with conventional oncologic workflows and ensure ethical outpatient implementation.

The first safety domain involved thyroidal responsiveness. Even localized molecular iodine exposure may transiently influence individuals with autoimmune thyroiditis, subclinical hypothyroidism, or altered iodine sensitivity. To minimize this risk, baseline thyroid-function assessment—including thyroid-stimulating hormone (TSH) and anti-thyroid peroxidase (anti-TPO) evaluation—was recommended together with short-term monitoring for potential Wolff–Chaikoff-like or paradoxical thyroidal responses (129, 130).

The second safety domain involved preservation of diagnostic timing and oncologic compatibility through short-interval imaging reassessment. A bilateral breast magnetic resonance imaging (MRI) checkpoint was systematically incorporated after the sixth outpatient session (approximately week three) as a predefined safety and response-oriented operational safeguard. This intermediate reassessment allowed early identification of limited responders and ensured that physiological modulation would not delay standard diagnostic escalation pathways such as fine-needle aspiration (FNA) or core-biopsy investigation (128).

The third safety domain involved dermal tolerability. Although overall compatibility was favorable, repeated exposure to pharmaceutical-grade dimethyl sulfoxide (DMSO; 99.9%) may occasionally induce mild transient irritation, erythema, warmth, or localized dryness (131). Likewise, superficial iodine-related pigmentation may occur following repeated topical exposure (132, 133). Within the present outpatient implementation pathway, these effects remained mild, reversible, and operationally manageable through isotonic saline pre-moistening of the application pad without compromising protocol feasibility or physiological responsiveness. No participant discontinued the intervention because of intolerance or adverse effects. Cases 1 and 2 completed the planned ten-session regimen, whereas the intervention was discontinued in Case 3 after the sixth session according to the predefined imaging-based stopping rule rather than because of tolerability or adherence limitations.

Collectively, these safety measures—including thyroidal monitoring, predefined MRI reassessment, and dermal-protection strategies—supported the construction of a controlled outpatient implementation environment capable of balancing physiological innovation with diagnostic safety, ethical compatibility, and operational reproducibility.

### 5.5 Translational exploratory outlook

The mechanistic convergence observed during the exploratory outpatient implementation suggests that the physiological principles underlying the iodine–DMSO protocol may extend beyond breast tissue and potentially interact with broader nodular conditions characterized by redox imbalance, endocrine responsiveness, stromal remodeling, and microenvironmental dysregulation.

Both breast and thyroid tissues exhibit high iodide avidity, overlapping hormonal receptor networks, and convergent oxidative-regulatory pathways, which may partially explain their recognized epidemiologic and endocrine co-dysregulation patterns (64, 134). Contemporary evidence further indicates that iodine-related physiological modulation extends beyond classical thyroid biology and may influence broader redox-regulatory and antiproliferative mechanisms (29). These shared biological characteristics support the theoretical relevance of exploring whether physiology-guided modulation strategies could demonstrate applicability across additional hormone-responsive nodular contexts.

Similarly, uterine fibroids and benign prostatic hyperplasia (BPH) represent nodular conditions influenced by intersecting pathways involving steroid signaling, oxidative stress, extracellular-matrix remodeling, stromal regulation, and perfusion heterogeneity (135–139). Developmentally, both uterine and prostatic tissues arise from hormonally responsive embryologic structures characterized by complex stromal–epithelial interactions and persistent endocrine sensitivity (140, 141). Contemporary evidence further demonstrates that extracellular-matrix remodeling, oxidative-stress signaling, inflammatory pathways, and microenvironmental dysregulation play central roles in the maintenance and progression of these nodular systems (142–144). Recent multi-omics and translational investigations therefore reinforce the presence of redox–immune–metabolic convergence patterns potentially associated with tissue-level physiological responsiveness across these hormonally regulated nodular contexts.

Taken together, these convergent biological features suggest that the regulatory axes explored within the iodine–DMSO outpatient framework—including redox modulation, tissue accessibility, perfusion responsiveness, and microenvironmental stabilization—may theoretically possess broader translational relevance across additional nodular systems characterized by comparable physiological organization, consistent with broader microenvironment-centered models of tissue regulation and nodular behavior (145).

At the same time, important translational limitations must be emphasized. Tissues such as vaginal and rectal mucosa possess markedly higher permeability and vascularity than dermal surfaces due to the absence of a stratum corneum and the presence of dense subepithelial microvasculature (146–149). Consequently, direct extrapolation of the present outpatient transdermal configuration to mucosal environments may substantially increase the risk of excessive absorption, local irritation, osmotic imbalance, or unpredictable systemic exposure—particularly in the presence of permeation-enhancing agents such as DMSO (150).

Although the human toxicology profile of DMSO has been extensively investigated under systemic and clinical exposure conditions (151), the markedly distinct permeability characteristics of mucosal tissues would still require cautious translational reinterpretation before any extension of the present outpatient framework beyond dermal application contexts.

For this reason, any future translational expansion involving mucosal or gland-specific applications would require dedicated preclinical validation incorporating *ex vivo* permeability studies, pharmacokinetic modeling, concentration-threshold evaluation, and safety-oriented delivery redesign (152).

Tables 12, 13 summarize the principal tissue-level convergences and integrative biological axes potentially relevant to future exploratory physiology-guided modulation research.

**Table 12:** Tissue-Level biological convergences potentially relevant to physiology-guided nodular modulation.

Tissue/System	Translationally Relevant Biological Features	Supporting References
Breast tissue (mammary epithelium and stroma)	High ER/PR receptor density; redox-sensitive proliferative behavior; nodular responsiveness influenced by perfusion gradients and vascular heterogeneity; epidemiologic convergence with thyroid dysregulation.	Winder et al. (2022) (29); Smyth (2016) (64).
Thyroid gland	Shared developmental relationship with foregut endoderm; iodine-dependent redox regulation; TR–ER signaling crosstalk; oxidative-regulatory pathways partially overlapping with breast tissue physiology.	Winder et al. (2022) (29); Smyth (2016) (64).
Uterine fibroids (leiomyomas)	Estrogen-responsive fibro-muscular nodular architecture associated with hypoxia-amplified redox imbalance, extracellular-matrix accumulation, and perfusion heterogeneity.	Islam et al. (2013) (135); Markowska et al. (2015) (136).
Benign prostatic hyperplasia (BPH)	Stromal–epithelial dysregulation associated with androgen/estrogen imbalance, oxidative-stress sensitivity, and microvascular remodeling dynamics.	Ho & Habib (2011) (138); Kaltsas et al. (2025) (139).

**Table 13:** Integrative biological axes potentially supporting future protocol expansion.

Integrative Axis	Translational Relevance	Supporting References
Nuclear hormone-receptor crosstalk	ER, PR, AR, and TR interactions may regulate transcriptional activity across breast, thyroid, uterine, and prostatic nodular contexts, supporting the relevance of endocrine-responsive modulation strategies.	Islam et al. (2013) (135); Ho & Habib (2011) (137).
Redox-sensitive gene expression	Convergence around Nrf2, HIF-1*α*, and oxidative-stress-responsive transcriptional programs may influence nodular persistence, remodeling, and physiological responsiveness.	Winder et al. (2022) (29); Markowska et al. (2015) (136).
Perfusion-driven metabolic gradients	Oxygen and nutrient-diffusion constraints may shape tissue-level nodular physiology and influence responsiveness to local modulation strategies across hormonally responsive tissues.	Islam et al. (2013) (135); Ho & Habib (2011) (138).
Stromal remodeling and extracellular-matrix stiffness	Shared extracellular-matrix accumulation, fibroblast–matrix interactions, and stromal tension may contribute to nodular persistence and heterogeneous tissue responsiveness.	Islam et al. (2013) (135); Kaltsas et al. (2025) (139).
Microenvironmental modulation	Ionic, redox, inflammatory, and perfusion gradients may act as shared determinants of nodular maintenance, tissue accessibility, and response-oriented physiological modulation.	Winder et al. (2022) (29); Cao et al. (2024) (144).

Collectively, these observations position the iodine–DMSO outpatient framework not only as a localized exploratory intervention model, but also as a broader translational platform for investigating response-oriented physiological modulation across hormonally responsive and microenvironmentally regulated nodular conditions.

Taken together, these exploratory findings position the iodine–DMSO outpatient framework not only as a localized physiology-guided intervention model, but also as a translational platform capable of informing future investigations into hormonally responsive and microenvironmentally regulated nodular conditions. By integrating mechanistic plausibility, operational reproducibility, imaging-based functional responsiveness, and structured outpatient implementation, the proposed protocol establishes a reproducible translational pathway connecting physiology-guided modulation with real-world clinical applicability.

## 6 Discussion

The present study positions the iodine–DMSO transdermal protocol as an exploratory translational proof-of-concept supported by convergent mechanistic evidence, structured protocol development, operational feasibility, and clinically observable imaging-response patterns. Through the sequential integration of mechanistic evidence synthesis, translational prioritization, outpatient protocol structuring, and exploratory clinical implementation, the proposed workflow evolved into a physiology-guided outpatient intervention model combining biological plausibility, operational reproducibility, and response-oriented imaging assessment within the management of BI-RADS 2–4B breast nodules.

Rather than functioning exclusively as a localized topical intervention, the protocol operated as a structured translational pathway integrating mechanistic convergence, tissue-level physiological modulation, outpatient feasibility, and short-interval functional reassessment. This integrative architecture allowed mechanistic findings related to redox regulation, tissue accessibility, perfusion responsiveness, and microenvironmental modulation to be progressively translated into an operational outpatient framework compatible with conventional oncologic workflows.

The exploratory imaging outcomes further suggest that physiology-guided outpatient modulation may generate measurable tissue-level responses within short intervals, potentially supporting dynamic decision-making strategies that extend beyond passive surveillance alone. Within this perspective, the protocol's translational relevance derives not only from the biological effects associated with iodine and DMSO co-intervention, but also from the structured outpatient implementation logic through which these mechanisms were operationalized.

### 6.1 Mechanistic convergence and physiological modulation in iodine-responsive breast tissue

The cumulative mechanistic evidence synthesized throughout the translational workflow supports the interpretation that iodine-responsive physiological modulation depends on the convergence of interconnected redox, endocrine, vascular, immune, and microenvironmental mechanisms rather than isolated intracellular signaling pathways alone. The systematic reinterpretation of iodine-related physiology demonstrated that molecular iodine exerts biologically coherent effects across apoptotic induction, epigenetic remodeling, endocrine responsiveness, and immune-related modulation domains (60–63).

Importantly, these biological effects appeared strongly conditioned by broader microenvironmental permissiveness involving oxygen diffusion, perfusion heterogeneity, ionic stability, stromal organization, and tissue-level redox balance (78–80). Evidence associated with NIS regulation, redox-sensitive signaling pathways, and oxidative-regulatory dynamics further reinforces the interpretation that iodine responsiveness may depend not only on iodine exposure itself, but also on the tissue-level physiological conditions governing uptake, diffusion, and intracellular functional expression (29, 71, 73).

Within this mechanistic architecture, dimethyl sulfoxide (DMSO) demonstrated translational relevance not merely as a dermal carrier, but as a biologically active co-adjuvant capable of reinforcing several of the mechanistic conditions associated with iodine-responsive modulation. Experimental and translational evidence synthesized in the present study demonstrated that DMSO may contribute to oxidative buffering, membrane permeability enhancement, perfusion responsiveness, stromal accessibility, and tissue-level physiological stabilization (109, 114, 121, 124).

These findings suggest that the observed translational convergence between iodine and DMSO extends beyond simple topical compatibility and may instead reflect a coordinated interaction between redox-sensitive modulation, tissue accessibility, and microenvironmental responsiveness. More broadly, structured drug-information resources and established frameworks for evaluating drug combinations provide complementary methodological foundations for characterizing pharmacological properties and interaction patterns during translational development (159, 160). From this perspective, iodine appears to provide biological directionality, while DMSO may facilitate the physicochemical and tissue-level conditions necessary for that physiological directionality to become operationally expressible within outpatient modulation contexts.

The exploratory outpatient observations obtained in WP6 were consistent with this mechanistic interpretation. The short-interval imaging-response patterns identified during protocol implementation—including contrast-enhancement reduction, vascular-signal modulation, stromal remodeling, and differential responsiveness trajectories—were consistent with several mechanistic domains predicted during the translational synthesis stages. Although preliminary, these findings support the broader hypothesis that physiology-guided modulation strategies may induce measurable tissue-level alterations within BI-RADS 2–4B nodular environments when mechanistic plausibility, tissue accessibility, and operational feasibility converge within a structured outpatient framework.

### 6.2 Functional responsiveness and response-oriented outpatient decision-making

One of the most relevant translational observations emerging from the exploratory outpatient implementation was the potential role of functional responsiveness as a dynamic imaging-based indicator capable of supporting short-interval clinical decision-making within BI-RADS 2–4B breast nodules. Rather than relying exclusively on static morphological classification, the proposed workflow incorporated sequential physiological reassessment to evaluate whether measurable tissue-level modulation could be detected during outpatient implementation.

The imaging-response patterns observed across the exploratory cases suggest that short-interval physiological modulation may generate clinically interpretable response trajectories associated with tissue remodeling, vascular normalization, and altered microenvironmental behavior. In Cases 1 and 2, the combination of contrast-enhancement reduction, Doppler-signal extinction, stromal normalization, and sustained remodeling during follow-up was compatible with the mechanistic domains previously associated with redox stabilization, perfusion responsiveness, and tissue-level physiological modulation.

Equally important was the interpretation of limited responsiveness. In the partial-response case, persistence of residual vascular signaling and incomplete imaging normalization during the predefined reassessment phase triggered immediate diagnostic escalation according to conventional oncologic workflows (128). This operational behavior illustrates an important conceptual distinction: within the proposed outpatient framework, imaging responsiveness does not function exclusively as an imaging outcome, but also as a response-oriented translational signal capable of informing whether outpatient modulation should continue or whether tissue-level uncertainty persists sufficiently to justify cytological clarification.

This interpretation aligns with growing interest in dynamic imaging reassessment strategies for indeterminate breast lesions and with broader evidence indicating that microenvironmental and redox-related dynamics may influence nodular evolution and imaging behavior (12, 15, 145). Within this context, short-interval imaging responsiveness may represent a clinically useful complement to conventional surveillance by introducing a functional dimension into outpatient breast-nodule management.

Importantly, the proposed workflow does not seek to replace biopsy, oncologic vigilance, or standardized BI-RADS-based decision pathways. Instead, it introduces an intermediate physiology-guided outpatient layer capable of dynamically identifying adaptive versus persistent tissue-level response patterns before escalation to invasive diagnostic procedures. This distinction is central to the translational rationale of the protocol because it positions outpatient physiological modulation not as an alternative to oncologic medicine, but as a structured response-oriented adjunct integrated within established diagnostic workflows.

From an implementation perspective, this approach may possess particular relevance in low-complexity ambulatory settings where prolonged surveillance, repeated short-interval imaging, and diagnostic uncertainty generate cumulative emotional, operational, and economic burden. By integrating physiological modulation with predefined safety checkpoints and response-oriented reassessment, the protocol establishes a translational outpatient pathway capable of linking tissue-level responsiveness with real-world clinical decision-making.

### 6.3 Translational implementation challenges and value-based outpatient innovation

Beyond its exploratory clinical observations, the present study also highlights broader translational challenges associated with implementing low-complexity, physiology-guided outpatient interventions within contemporary breast-care systems. Although substantial mechanistic evidence supported the biological plausibility of iodine-responsive modulation and the outpatient implementation pathway demonstrated operational feasibility, the translational positioning of interventions situated between passive surveillance and conventional oncologic escalation remains structurally complex within evidence-hierarchy-driven clinical environments.

Current breast-care workflows are strongly optimized around imaging surveillance, biopsy confirmation, and escalation-oriented diagnostic pathways. Consequently, low-risk physiology-guided interventions designed to function as short-interval response-oriented outpatient layers may occupy an intermediate translational space that is not fully encompassed by conventional therapeutic or diagnostic classifications. This creates important implementation challenges because mechanistic convergence, operational feasibility, and outpatient usability do not always align with the traditional evidentiary structures through which novel breast-care interventions are typically evaluated.

Within this context, the iodine–DMSO outpatient protocol demonstrates a translational profile that differs substantially from high-complexity oncologic innovations. The intervention does not depend on proprietary technologies, advanced procedural infrastructure, or highly specialized delivery systems. Instead, it operates through a low-cost outpatient architecture integrating short-interval physiological modulation, predefined safety checkpoints, and imaging-based functional reassessment. This operational simplicity may represent both a translational advantage and an implementation challenge, particularly in clinical environments historically oriented toward high-complexity escalation pathways.

These considerations possess direct relevance within Value-Based Healthcare (VBHC) perspectives emphasizing outcome optimization, reduction of unnecessary procedural burden, and operational sustainability159,160. In the present workflow, the protocol functioned simultaneously as a physiology-guided modulation strategy and as a response-oriented outpatient triage layer capable of dynamically identifying lesions demonstrating adaptive versus persistent tissue-level responsiveness. Such dual functionality may contribute to reducing prolonged uncertainty, repeated imaging cycles, and unnecessary escalation in selected BI-RADS 2–4B contexts while preserving oncologic vigilance.

The economic implications of this translational positioning may be particularly relevant in resource-sensitive healthcare systems, especially considering the growing global burden associated with breast-cancer diagnostic pathways (153). Global evidence demonstrates that financial toxicity associated with breast-cancer care extends beyond therapeutic expenditure and begins during prolonged diagnostic trajectories characterized by repeated imaging, specialist consultations, and invasive procedures (154). Because the proposed outpatient workflow operates through low-cost materials, minimal procedural infrastructure, short exposure windows, and ambulatory implementation logic, even modest reductions in unnecessary diagnostic escalation could theoretically generate meaningful operational and economic impact within high-volume surveillance settings.

Importantly, the translational relevance of the present intervention does not derive solely from the biological activity of iodine and DMSO themselves, but from the structured outpatient implementation pathway through which mechanistic plausibility, operational reproducibility, safety checkpoints, and response-oriented reassessment were integrated. This distinction reinforces the broader concept that low-complexity translational innovations may acquire clinical relevance not through isolated mechanistic novelty alone, but through their capacity to operationalize biologically coherent strategies within scalable outpatient environments. This progression is consistent with established conceptualizations of translational research as a continuum connecting mechanistic discovery, clinical investigation, and real-world implementation, while addressing the persistent challenge of bridging the gap between basic and clinical research (161–163).

Within this perspective, the present study illustrates how physiology-guided outpatient interventions may contribute to expanding the translational repertoire available for managing indeterminate breast nodules by introducing dynamic functional reassessment into environments traditionally dominated by static surveillance paradigms. Although exploratory and preliminary, the findings support the broader hypothesis that structured outpatient modulation strategies may possess both physiological and implementation-oriented relevance within future VBHC-compatible breast-care models.

This translational positioning also highlights a broader implementation challenge frequently encountered by low-complexity physiology-guided interventions situated between conventional surveillance and definitive oncologic escalation. Although mechanistically coherent and operationally feasible, such outpatient strategies often occupy an intermediate translational space that does not align neatly with traditional categories of pharmacologic therapy, device-based intervention, or purely diagnostic innovation. As a consequence, interventions emphasizing physiological modulation, operational simplicity, and response-oriented outpatient reassessment may experience substantial translational friction despite presenting biologically plausible clinically relevant implementation pathways.

This challenge reflects a broader structural characteristic of contemporary evidence ecosystems, in which interventions supported by mechanistic convergence, outpatient feasibility, and usability-oriented implementation may still face difficulty achieving rapid incorporation into conventional specialty-oriented translational pathways when large-scale randomized validation and industry-driven development structures are absent. Within breast-care environments strongly organized around surveillance and escalation models, the integration of low-cost physiology-guided outpatient strategies may therefore require not only clinical validation, but also conceptual adaptation regarding how translational relevance is operationally interpreted.

### 6.4 Scalability, operational simplicity, and outpatient feasibility

One of the most distinctive translational characteristics of the iodine–DMSO outpatient framework is its intrinsic scalability profile. Unlike many contemporary breast-care innovations that depend on proprietary technologies, advanced imaging platforms, or highly specialized procedural infrastructure, the proposed intervention operates through a low-complexity outpatient architecture based on short-duration transdermal exposure, standardized monitoring checkpoints, and response-oriented imaging reassessment.

The protocol requires only pharmaceutical-grade iodine and DMSO, sterile disposable materials, basic outpatient supervision, and conventional imaging support already routinely available within breast-care pathways. The intervention does not depend on surgical infrastructure, complex device integration, or advanced procedural training, allowing implementation within general outpatient clinics, gynecological services, and ambulatory breast-care environments.

This operational simplicity possesses important translational implications. Because the protocol can be executed within short outpatient sessions using minimal infrastructure, its marginal implementation cost remains low while preserving compatibility with conventional oncologic workflows. In addition, the standardized application sequence and reproducible monitoring structure facilitate operational scalability across environments with different levels of technological complexity.

These characteristics may possess particular relevance within resource-sensitive healthcare systems where prolonged surveillance pathways, repeated imaging reassessment, and invasive diagnostic escalation generate cumulative operational and economic burden. Contemporary evidence further demonstrates that breast-cancer care is associated with substantial direct, indirect, and humanistic costs, particularly when diagnostic and therapeutic pathways become prolonged or operationally complex (155). Within such contexts, low-cost physiology-guided outpatient strategies capable of supporting dynamic functional reassessment may contribute to reducing unnecessary diagnostic repetition while preserving clinical vigilance.

Importantly, the scalability of the present framework derives not only from material simplicity, but also from its operational logic. The protocol integrates predefined safety checkpoints, reproducible implementation sequences, and response-oriented reassessment criteria capable of maintaining diagnostic compatibility during outpatient modulation. This combination of mechanistic plausibility, operational reproducibility, and implementation simplicity distinguishes the protocol from many exploratory translational interventions that remain difficult to operationalize beyond specialized research environments.

From a Value-Based Healthcare (VBHC) perspective (156, 157), these implementation characteristics reinforce the possibility that low-complexity physiology-guided outpatient interventions may contribute simultaneously to operational sustainability, reduction of procedural burden, and enhancement of patient-centered decision-making. Although formal cost-effectiveness analyses remain necessary, the present outpatient architecture already incorporates several structural elements commonly associated with scalable healthcare innovation, including low infrastructural dependence, workflow reproducibility, outpatient compatibility, and response-oriented implementation logic.

### 6.5 Limitations and future directions

Several limitations must be acknowledged when interpreting the present exploratory findings. First, the outpatient implementation involved a limited number of clinical cases without a control group, preventing definitive conclusions regarding efficacy, durability, or comparative clinical performance. The imaging outcomes observed during short-interval reassessment therefore should be interpreted as exploratory translational signals rather than as confirmatory therapeutic endpoints. Given the uncontrolled exploratory design, spontaneous evolution of benign lesions and variability inherent to imaging assessment cannot be excluded as alternative contributors to the observed imaging-response patterns.

Second, although the observed imaging-response patterns were mechanistically coherent with the physiological domains identified throughout the translational synthesis, direct molecular confirmation through biomarker-oriented analyses was not performed. Future investigations incorporating cytokine profiling, oxidative-stress indices, NIS-expression assessment, endocrine-response markers, and microenvironment-related signaling analyses may further clarify the biological pathways associated with iodine–DMSO responsiveness.

Third, the current implementation pathway evaluated short-interval imaging-defined physiological modulation rather than histopathologically confirmed lesion resolution. Histopathological confirmation was not available for Cases 1 and 2, in which complete radiologic resolution was observed; consequently, radiologic resolution should not be interpreted as histopathological proof of lesion eradication or as confirmatory evidence of therapeutic efficacy. Although Case 1 demonstrated sustained imaging stability during a 12-month follow-up period, broader longitudinal validation remains necessary to determine durability, recurrence patterns, and long-term outpatient applicability across heterogeneous nodular contexts.

Additional limitations relate to translational generalizability. Because the protocol was implemented within a structured outpatient environment incorporating predefined imaging checkpoints, standardized application procedures, and institutional oversight, extrapolation to unsupervised or non-standardized settings should be approached cautiously. Moreover, although the broader translational framework encompasses BI-RADS 2–4B nodules, prospective exploratory implementation was restricted to three BI-RADS 4B cases, two of which also presented contralateral BI-RADS 2 nodules; broader category-specific generalization therefore requires further evaluation. Likewise, the exploratory translational outlook regarding additional hormonally responsive nodular systems remains theoretical and requires dedicated mechanistic and preclinical validation before any clinical expansion can be considered.

Despite these limitations, the present study establishes several relevant directions for future investigation. Prospective controlled outpatient studies incorporating larger cohorts, standardized imaging-response metrics, and biomarker-oriented monitoring may help determine whether short-interval physiology-guided modulation can reproducibly contribute to outpatient decision-making within BI-RADS 2–4B management pathways. Future investigations may also evaluate whether structured integration with broader systemic physiological optimization strategies could improve responsiveness in partially responsive nodular environments.

From an implementation-science perspective, future work should additionally investigate reproducibility across different outpatient infrastructures, scalability under real-world clinical conditions, patient-centered outcomes, workflow integration, and formal cost-effectiveness modeling. Such investigations may help determine whether low-complexity physiology-guided modulation strategies can evolve from exploratory translational interventions into reproducible outpatient tools compatible with broader Value-Based Healthcare implementation frameworks.

## 7 Conclusions

The present study establishes a structured physiology-guided outpatient framework for the management of BI-RADS 2–4B breast nodules through the integration of mechanistic evidence synthesis, translational prioritization, operational protocol structuring, and exploratory clinical implementation. The exploratory implementation of the iodine–DMSO transdermal protocol yielded biologically coherent, imaging-detectable, and operationally reproducible response patterns compatible with short-interval outpatient physiological modulation.

Across the exploratory outpatient implementation pathway, measurable imaging-response trajectories were observed in association with tissue remodeling, vascular-signal modulation, and functional responsiveness while preserving compatibility with conventional oncologic workflows through predefined safety checkpoints and response-oriented reassessment strategies. These observations support the broader interpretation that physiology-guided outpatient modulation may function not only as a low-complexity intervention strategy, but also as a dynamic translational adjunct capable of contributing to short-interval decision-making within indeterminate breast-nodule management.

Importantly, the translational relevance of the proposed framework derives not solely from the biological activity associated with iodine and DMSO co-intervention, but from the structured outpatient implementation architecture through which mechanistic plausibility, tissue accessibility, imaging responsiveness, operational reproducibility, and safety-oriented monitoring were integrated into a coherent ambulatory workflow.

The operational simplicity of the protocol—including low infrastructural dependence, standardized transdermal application, short-duration outpatient exposure, and compatibility with existing imaging pathways—further reinforces its potential translational applicability within low-complexity and resource-sensitive healthcare environments. In this context, the protocol aligns with broader Value-Based Healthcare principles (156, 157) by introducing a response-oriented outpatient strategy potentially capable of reducing prolonged diagnostic uncertainty, unnecessary escalation, and cumulative procedural burden while preserving oncologic vigilance.

Although exploratory and limited by sample size and absence of controlled comparison, the findings support further investigation of structured physiology-guided outpatient modulation strategies as a potential complement to passive surveillance, incorporating dynamic imaging reassessment and functional responsiveness evaluation. Future controlled investigations incorporating larger cohorts, biomarker-oriented monitoring, longitudinal follow-up, and implementation-science analyses will be essential to determine the reproducibility, scalability, and broader clinical applicability of the proposed framework.

Taken together, the present study establishes the iodine–DMSO outpatient protocol as a biologically plausible, operationally reproducible, and translationally structured intervention pathway capable of connecting mechanistic rationale with real-world ambulatory implementation for further investigation within BI-RADS 2–4B breast-nodule management.

## Data Availability

The data supporting the findings of this study are contained within the article. The systematic review components were prospectively registered in PROSPERO under registration numbers CRD420251122511 and CRD420251123805. No supplementary datasets were generated or deposited in an external repository.
